# Titin Circular RNAs Create a Back-Splice Motif Essential for SRSF10 Splicing

**DOI:** 10.1161/CIRCULATIONAHA.120.050455

**Published:** 2021-02-15

**Authors:** Anke J. Tijsen, Lucía Cócera Ortega, Yolan J. Reckman, Xiaolei Zhang, Ingeborg van der Made, Simona Aufiero, Jiuru Li, Selina C. Kamps, Anouk van den Bout, Harsha D. Devalla, Karin Y. van Spaendonck-Zwarts, Stefan Engelhardt, Lior Gepstein, James S. Ware, Yigal M. Pinto

**Affiliations:** 1 Amsterdam UMC, University of Amsterdam, Departments of Experimental Cardiology, Amsterdam Cardiovascular Sciences (A.J.T., L.C.O., Y.J.R., I.v.d.M., S.A., S.C.K., A.v.d.B., Y.M.P.), Amsterdam, The Netherlands.; 2 Medical Biology, Amsterdam Cardiovascular Sciences (J.L., H.D.D.), Amsterdam, The Netherlands.; 3 Clinical Genetics (K.Y.v.S.-Z.), Amsterdam, The Netherlands.; 4 Imperial College London, South Kensington Campus, London, UK (X.Z., J.S.W.).; 5 DZHK (German Center for Cardiovascular Research), partner site Munich Heart Alliance, Munich, Germany (S.E.).; 6 Institut für Pharmakologie und Toxikologie, Technische Universität München, Munich, Germany (S.E.).; 7 The Sohnis Family Laboratory for Cardiac Electrophysiology and Regenerative Medicine, Rappaport Faculty of Medicine and Research Institute, Technion-Institute of Technology, Haifa, Israel (L.G.).

**Keywords:** alternative splicing, cardiomyopathies, induced pluripotent stem cells, RNA, circular, RNA, untranslated, SRSF10 protein, human

## Abstract

**Background:**

TTN (Titin), the largest protein in humans, forms the molecular spring that spans half of the sarcomere to provide passive elasticity to the cardiomyocyte. Mutations that disrupt the *TTN* transcript are the most frequent cause of hereditary heart failure. We showed before that *TTN* produces a class of circular RNAs (circRNAs) that depend on RBM20 to be formed. In this study, we show that the back-splice junction formed by this class of circRNAs creates a unique motif that binds SRSF10 to enable it to regulate splicing. Furthermore, we show that one of these circRNAs (cTTN1) distorts both localization of and splicing by RBM20.

**Methods:**

We calculated genetic constraint of the identified motif in 125 748 exomes collected from the gnomAD database. Furthermore, we focused on the highest expressed RBM20-dependent circRNA in the human heart, which we named cTTN1. We used shRNAs directed to the back-splice junction to induce selective loss of cTTN1 in human induced pluripotent stem cell–derived cardiomyocytes.

**Results:**

Human genetics suggests reduced genetic tolerance of the generated motif, indicating that mutations in this motif might lead to disease. RNA immunoprecipitation confirmed binding of circRNAs with this motif to SRSF10. Selective loss of cTTN1 in human induced pluripotent stem cell–derived cardiomyocytes induced structural abnormalities, apoptosis, and reduced contractile force in engineered heart tissue. In line with its SRSF10 binding, loss of cTTN1 caused abnormal splicing of important cardiomyocyte SRSF10 targets such as *MEF2A* and *CASQ2*. Strikingly, loss of cTTN1 also caused abnormal splicing of *TTN* itself. Mechanistically, we show that loss of cTTN1 distorts both localization of and splicing by RBM20.

**Conclusions:**

We demonstrate that circRNAs formed from the *TTN* transcript are essential for normal splicing of key muscle genes by enabling splice regulators RBM20 and SRSF10. This shows that the *TTN* transcript also has regulatory roles, besides its well-known signaling and structural function. In addition, we demonstrate that the specific sequence created by the back-splice junction of these circRNAs has important functions. This highlights the existence of functionally important sequences that cannot be recognized as such in the human genome but provides an as-yet unrecognized source for functional sequence variation.

## Abstract 2

Supplemental Digital Content is available in the text.

**Clinical Perspective**

**What Is New?**

- We uncovered the large, heart-specific circular RNA cTTN1, which is derived from the I-band region of the *TTN* (Titin) transcript and forms a functional motif when both ends of the RNA join on circularization.
- cTTN1 is necessary for normal cardiomyocyte organization, and we show that the motif that this and other RBM20-dependent circular RNAs form by circularization is crucial to allow binding of SRSF10 to exert its function.
- This reveals that the *TTN* transcript forms functionally important circular RNAs, and together with our finding that genomic regions corresponding to motif formation are less tolerant to sequence variation this suggests that variants that affect formation and function of these circRNAs might relate to cardiac pathology.

**What Are the Clinical Implications?**

- Besides mutations in *RBM20*, mutations in the I-band region of the *TTN* gene also may have as-yet unsuspected clinical relevance by hindering formation or function of these circular RNAs.
- In particular, variants in this region may have more clinical relevance than was assumed on the basis of the role of this region in the formation of the TTN protein.

The human titin (*TTN*) gene consists of 363 exons to produce the largest human protein. It is regarded as a bidirectional spring that provides mechanical support to the sarcomere but also receives incoming signals.^1^ With the advent of next-generation sequencing, it became clear that truncating variants in *TTN* are found in 20% to 30% of patients with a dilated cardiomyopathy (DCM).^2^ While the role of TTN in cardiomyocyte function and structure is well established, far less is known about the possible regulatory roles of the RNA transcript (>100 kb) that *TTN* produces.

It was long assumed that in pre-mRNAs exons are always spliced to an upstream exon to generate a linear mRNA, which then serves to translate to protein. However, with the advent of next-generation sequencing, it became increasingly clear that many genes can also form circular RNAs (circRNAs) by so-called back-splicing.^3–5^ Back-splicing is a noncanonical form of splicing in which the donor sequence of an upstream exon covalently links to the acceptor sequence of a downstream exon (back-splice junction), which results in the formation of single-stranded circRNA molecules. Depending on the linear splicing in the template pre-mRNA, this circRNA might contain all or only a few of the exons between the acceptor and donor exons. Although for most of the circRNAs the function is still elusive, several functions have been described, and no general function seems applicable to all of them. The described functions range from microRNA sponging^4,5^ to facilitation of transcription by direct association with RNA polymerase II,^6^ to interaction with proteins,^7^ to acting as a template for protein synthesis.^8^

We and others have shown the expression of thousands of circRNAs in the human and rodent heart, where especially the mRNA transcripts of *RYR2* and *TTN* produce large numbers of circRNAs.^9–12^ In our study, we also showed that the splice regulator RBM20 is necessary to produce a large proportion of these *TTN*-derived circRNAs.^9^ This class of RBM20-dependent circRNAs is derived mainly from the I-band region of *TTN*, which is the region that is alternatively spliced in *RBM20* mutation carriers.^9,13,14^ This suggests that loss of these circRNAs in *RBM20* mutation carriers might contribute to the development of the severe form of DCM combined with lethal arrhythmias, but also that *TTN* mutation carriers in the I-band region could develop similar phenotypes as a result of the distortion of formation or function of these circRNAs. Therefore, we asked if this specific class of RBM20-dependent circRNAs formed from the I-band region of the *TTN* transcript have important functions in cardiomyocytes.

## Methods

Raw sequencing data for circRNA detection in human heart and cTTN1 inhibition in human induced pluripotent stem cell (hiPSC)–derived cardiomyocytes (hiPSC-CMs) are available via NCBI accession numbers PRJNA5333243 and PRJNA630157, respectively. All data with regard to bioinformatic analysis are included in Excel Files I through VI in the Data Supplement. Data of RNAseR-treated human heart tissue RNA sequencing (RNAseq) were used under license for the current study; data are available via the corresponding authors. CircRNAprofiler is available on github page: https://github.com/Aufiero/circRNAprofiler.^15^ The code for the genetic constraint analysis is available at https://github.com/ImperialCardioGenetics/cTTN.

Extended methods are described in the Data Supplement, which also includes primer and shRNA sequences and antibody details in Tables I, II, and III in the Data Supplement, respectively.

### hiPSC Culture and Differentiation

The fully characterized hiPSC line derived from a healthy man was previously published.^16^ The dermal fibroblasts were obtained with informed consent after approval by the Institutional Review Board committee of Rambam Medical Center. Colonies of these hiPSCs were cultured in mTeSR-1 on plates coated with growth factor–reduced Matrigel. Cardiac differentiation was performed following a previously published protocol^17^ with the slight adaptation that cells were cultured in RPMI/B27 medium from days 7 to 10. Cells were metabolically selected for at least 2 weeks by culturing in CDM3 medium without glucose supplemented with 20 mmol/L sodium lactate.^18^ All experiments were conducted on hiPSC-CMs 40 to 60 days after the start of differentiation, and each observation was replicated in 2 to 5 independent experiments with hiPSC-CMs from different differentiations.

### hiPSC-CM Infection

We cloned shRNAs targeting the back-splice junction of cTTN1 and a negative control shRNA (shSCR) into the pLKO.1 vector and produced third-generation lentivirus in RPMI/B27 or CDM3 medium, depending on the experiment. hiPSC-CMs were dissociated with the use of TrypLE express and replated 2 to 4 days before lentiviral transduction to ensure homogeneous cell populations between conditions. The hiPSC-CMs were transduced either with freshly produced lentivirus or with a specific amount of transducing units, depending on the density of the cells. Experiments were performed on cells between 4 and 8 days after transduction.

### RNA Experiments

RNA was isolated with TriReagent. RNAseq was performed on a Illumina NextSeq 500 platform in paired-end mode with a read length of 150 bp and analyzed by use of the R Bioconductor packages DEseq2 and DEXseq for differential gene expression and exon usage, respectively. For detection of most circRNAs, we used end-point polymerase chain reaction (PCR) with divergent primers to amplify the back-splice region, and for detection of cTTN1, we used a TaqMan-based quantitative PCR system with similar divergent primers and a probe targeting the back-splice junction. To visually detect cTTN1 in fixed cells, we used a FAM-labeled probe directed against the back-splice junction, where the signal was amplified by incubation with an anti-FITC antibody followed by incubation with a TSA Plus fluorescence kit. RNA immunoprecipitation was performed with the MagnaRIP RNA binding protein immunoprecipitation kit. RBM20 knockout (KO) mice hearts for RNAseq were derived from studies approved by the Institutional Animal Care and Use Committee of the University of Amsterdam and performed in accordance with the guidelines of this institution.

### Calculation of Genetic Constraint of Genomic Regions

We compared the observed number of rare variants (allele frequency <0.1%) in the gnomAD reference population (version 2.1.1) exome data sets (125 748 individuals) with the number of variants that we would expect to see under neutral variation. The expected number of variants under neutral selection was predicted based on sequence context and methylation level,^19^ and in a second step, the expected numbers were corrected for sequencing depth at low-coverage sites. The model was validated using *TTN* synonymous, missense, and truncating variants; the first 2 are assumed to be under minimal selection, and truncating variants are known to cause DCM.

### Bioinformatic Predictions

For prediction of RNA-binding protein and microRNA (miRNA) binding, we used the raw sequencing data of our previously published study.^9^ Here, we used the paired-end RNAseq reads of 3 human control hearts, 3 DCM hearts, and 3 hypertrophic cardiomyopathy hearts and aligned them against the human genome reference hg19 using MapSplice, CircMarker, and NCLscan. We predicted both the RNA-binding protein and miRNA binding using our in-house previously published R-based computation framework, circRNAprofiler.^15^

For bioinformatic prediction of exons included in cTTN1, we used RNAseq data of RNAseR-treated RNA samples isolated from left ventricular tissue and the computational tool circAST, which determines the exons derived from aligning of these sequencing data to the human genome and thus derived from circRNAs in a specific region of *TTN*.

### Statistical Analysis

Data obtained from hiPSC-CMs are a combination of 2 to 5 independent experiments on cells from independent differentiations, with at least n=2 biological replicates per independent experiment. Data from these independent experiments are combined by use of Factor Correction.^20^ As a consequence, the data shown for continuous variables are a mean±SEM of n=6 to 15 biological replicates derived from 2 to 5 differentiations. For categorical data, the percentage of cells in all groups is depicted per condition. To compare continuous variables between 2 groups, we used the Mann-Whitney *U* test; to compare continuous variables between 3 groups, we used the Kruskal-Wallis test combined with the Dunn post hoc test. To compare the distribution of cells in different categories between 2 groups, we used the χ^2^ test. To compare the effect of loss of RBM20 over time (days 1–8), we made use of a 2-way ANOVA to test the effect of loss of RBM20, the day effect, and the interaction term between RBM20 and day. In case the interaction term was significant, we determined the differences between the shRNA against *RBM20* and the negative control shRNA by pairwise comparison, in which we used Bonferroni correction for multiple testing. All performed tests were 2 sided, and a value of *P*<0.05 was considered significant.

## Results

### Functional Motif Created by Back-Splicing

**Figure 1.**
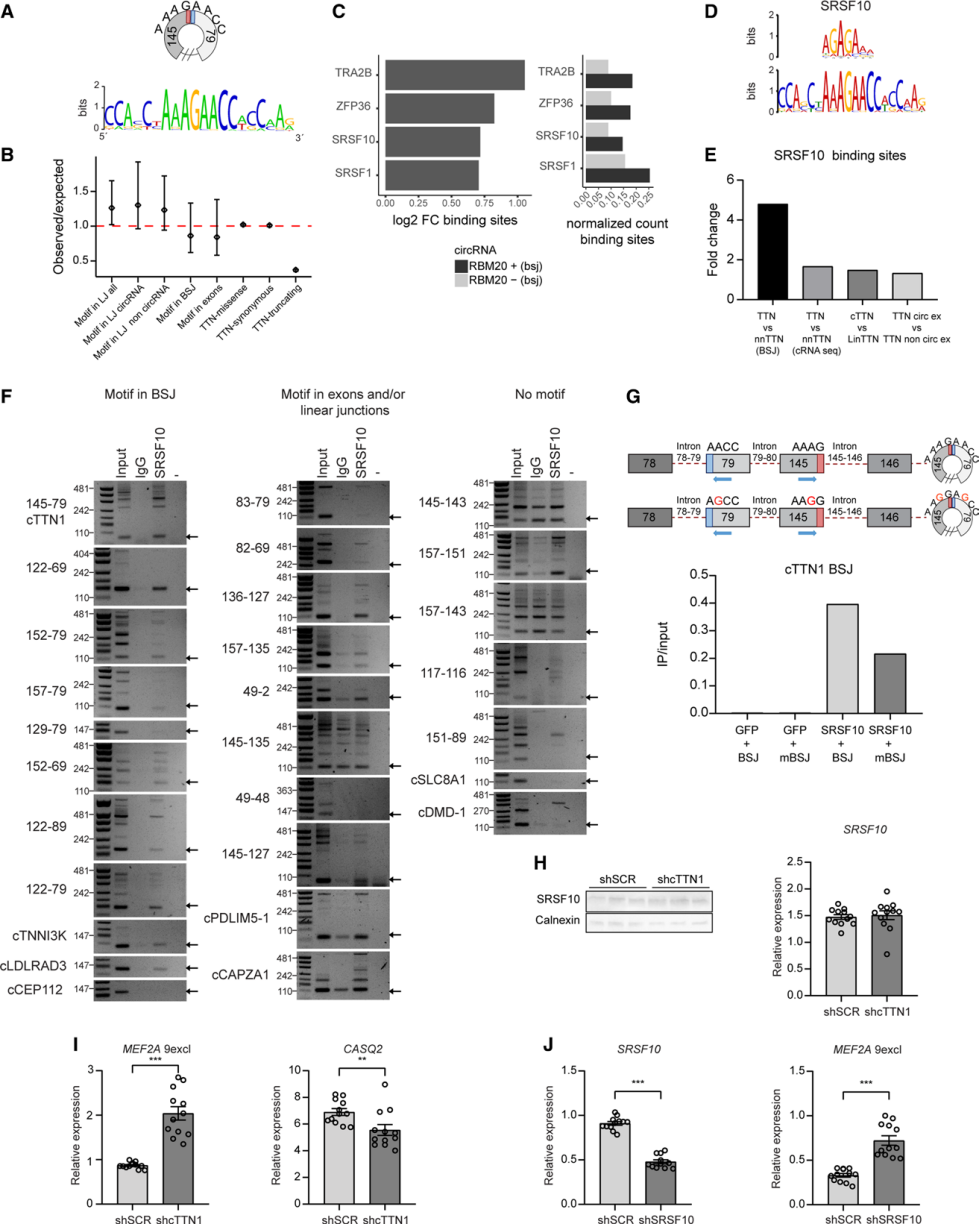
**Circularization of RBM20-dependent TTN (Titin)-derived circular RNAs (circRNAs) creates a functional SRSF10 binding site.** **A**, Alignment of RBM20-dependent *TTN*-derived circRNAs shows an 8-nucleotide motif created in the back-splice junction. **B**, Genetic constraint plot showing the ratio (with 90% CI) between observed and predicted variants under neutral mutation modeling for the motif created by linear splicing (LJ) in all *TTN* exons and separated for exons included or excluded from circRNAs, for the motif created by back-splicing (BSJ) and within exons. As control for the modeling *TTN* missense, synonymous and truncating variants are depicted. A ratio <1 indicates reduced genetic constraint. **C**, Comparison of detected RNA binding protein motifs within the back-splice junction between RBM20-dependent and -independent circRNAs using the ATtRACT database. **D**, MEME prediction of RNA binding proteins binding to the created back-splice junction motif in RBM20-dependent circRNAs. **E**, Comparison of detected SRSF10 motifs in back-splice junction and circRNA sequences of *TTN*-derived vs non–*TTN*-derived circRNAs, of circRNA sequences of *TTN*-derived circRNAs vs the linear *TTN* mRNA, and of *TTN* exons included vs not included in any circRNA. **F**, Reverse transcriptase–polymerase chain reaction (RT-PCR)–based detection of circRNAs with the motif in their back-splice junction, full circRNA sequence, or without any motif after immunoprecipitation based on endogenous SRSF10 in human induced pluripotent stem cell–derived cardiomyocytes (hiPSC-CMs; 3 repeats in Figure IV in the Data Supplement). **G**, Quantitative RT-PCR (qRT-PCR) of an artificial circRNA construct creating the motif or a mutated motif in the back-splice junction after SRSF10 immunoprecipitation in HEK293T cells. **H**, Western blot (n=3) and qRT-PCR for SRSF10 after knockdown of cTTN1 in hiPSC-CMs. **I** and **J**, qRT-PCR of *SRSF10* and its splice (MEF2A exclusion exon 9) and expression (*CASQ2*) targets after knockdown of cTTN1 (**I**) and *SRSF10* (**J**) in hiPSC-CMs. Error bars indicate SEM; qRT-PCRs n=12 from 4 differentiations. **P<0.025; ***P<0.001.

First, we looked for commonalities in the RBM20-dependent circRNAs. The most striking commonality was that 70% of RBM20-dependent circRNAs form a specific sequence in the back-splice junction when they circularize, which is the motif AAAGAACC (Figure 1A and Figure Ia and Ib in the Data Supplement). This motif occurs almost exclusively in the back-splice junction of RBM20-dependent circRNAs derived from *TTN*; it is found in only 3 other non-*TTN*-derived circRNAs, whereas it is found in 44 *TTN*-derived circRNAs. The biological importance of this motif is further suggested by the trend for selective constraint we found within the human population (Figure 1B).^19^ This genetic constraint is calculated by comparing the observed number of variants in 125 748 exomes collected from the gnomAD database with the expected number of variants within these exomes derived from neutral mutation modeling (Figure II in the Data Supplement).^21^ We clearly found no selective constraint when the above-described motif was formed by linear *TTN* splicing independently of whether this splicing occurred in exons included or always excluded from circRNAs. However, we did detect a trend toward reduced selective constraint within this motif when it is both formed by back-splicing and present within an exon (Figure 1B). This indicates that variation in the motif created by back-splicing is less well tolerated and could be associated with disease.

To further investigate the biological function of this motif, we used the ATtRACT and MEME databases (Figure 1C and 1D) to predict which RNA binding proteins could recognize this motif. Both approaches predicted that the motif can bind the splice regulator SRSF10 (*FUSIP1/SRp38*). Loss of *SRSF10* was shown previously to result in embryonic lethality attributable to an underdeveloped myocardium and distorted cardiomyocyte calcium handling.^22,23^ A second bioinformatic comparison using all annotated SRSF10 binding motifs in ATtRACT revealed an enrichment in the back-splice junction and the full sequence of *TTN*- versus non–*TTN*-derived circRNAs, in the full sequence of *TTN*-derived circRNAs versus the linear *TTN* mRNA, and in the exons included versus excluded from the circRNAs (Figure 1E and Figure III and Excel Files I and II in the Data Supplement). To test this predicted binding, we performed RNA immunoprecipitation of SRSF10 in hiPSC-CMs. Indeed, all tested circRNAs in which the back-splice junction forms the motif strongly bind SRSF10. In addition, circRNAs that contain the motif in their full sequence (ie, somewhere other than in their back-splice junction) also bind SRSF10. In contrast, circRNAs without the motif do not bind SRSF10 (Figure 1F and Figure IV in the Data Supplement). In addition, in a RNA immunoprecipitation in which we overexpressed SRSF10 and an artificial circRNA construct containing the motif, mutation of the motif impaired this binding (Figure 1G). Therefore, we concluded that when the back-splice junction of these circRNAs forms this specific motif, it enables SRSF10 to bind.

SRSF10 is a known regulator of splicing, which led us to surmise that loss of such a circRNA might impair the ability of SRFS10 to regulate splicing in the heart. To further analyze this, we focused on cTTN1, the human-specific circRNA that we found to be highest expressed in the heart and to be downregulated in patients with DCM.^9,10^ This circRNA is formed by back-splicing of exon 145 to exon 79 and is predicted to span 67 exons. We used the computational tool circAST^24^ on RNAseq data of RNAseR-treated myocardial RNA, which revealed that within these 67 exons, only 9 exons are not included in any circRNA from the I-band region, whereas 35 exons are detected in circRNAs in all 3 human hearts (Excel File III in the Data Supplement). This suggests that cTTN1 is very large and could potentially span 35 to 58 exons in total. We used shRNAs that target the back-splice junction of cTTN1 to selectively knock down cTTN1 without affecting its linear counterpart (Figure V in the Data Supplement). Loss of cTTN1 caused missplicing of SRSF10 targets such as *CASQ2* and *MEF2A* in the same way that loss of *SRSF10* does, without affecting *SRSF10* expression levels (Figure 1H–1J). Taken together, these findings demonstrate that the back-splice junction of cTTN1 creates a motif that binds SRSF10, which is essential to allow normal SRSF10 function.

**Figure 2.**
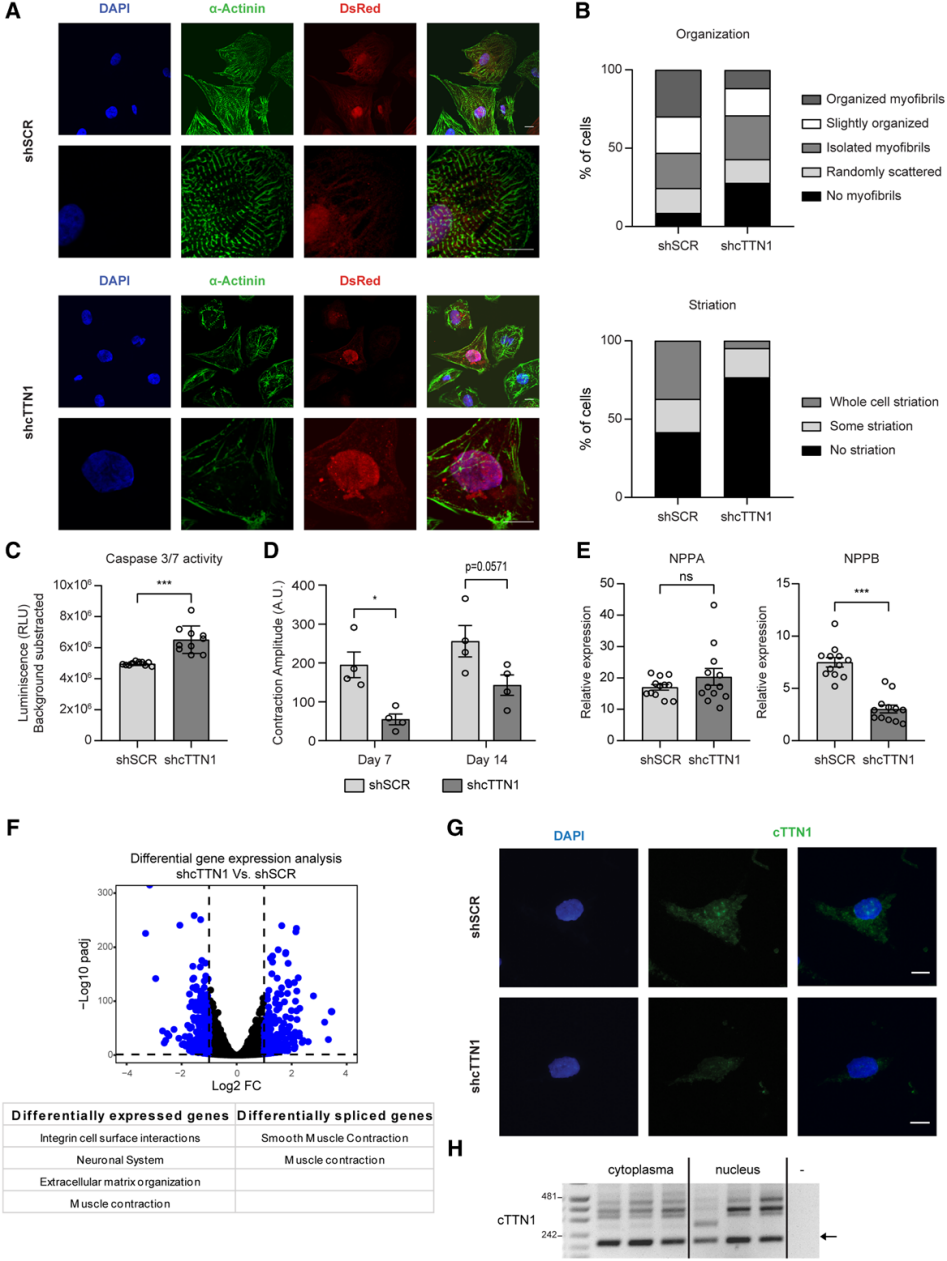
**Loss of cTTN1 results in disorganized sarcomeres and apoptosis.** **A**, Representative pictures at 2 magnifications of immunocytochemistry after loss of cTTN1 in human induced pluripotent stem cell–derived cardiomyocytes (hiPSC-CMs; (DAPI, blue; sarcomeric α-actinin, green; dsRED, red; scale bars, 10 μm). **B**, Quantification of **A** for sarcomere organization (0–4 from not present to aligned myofibrils) and striation (0–2 from not present to present in the whole cell). N=151 negative control shRNA (shSCR) and 86 shcTTN1 from 4 differentiations. *P*<0.001, χ^2^ test. **C**, Luciferase-based caspase 3/7 activity in hiPSC-CMs after loss of cTTN1 (n=10; 5 differentiations). **D**, Contraction amplitudes 7 and 14 days after generation of engineered heart tissues generated from hiPSC-CMs after loss of cTTN1 (n=4; 3 differentiations) **E**, Quantitative reverse transcriptase–polymerase chain reaction (qRT-PCR) of stress markers after loss of cTTN1 (n=12; 4 differentiations). **F**, Volcano plot representing differential gene expression based on RNA sequencing (n=3) after loss of cTTN1 with differentially expressed genes in blue, and table indicating the affected pathways based on differentially expressed genes or spliced exons. **G**, Representative images of cTTN1 fluorescent in situ hybridization in hiPSC-CMs (DAPI, blue; cTTN1, green; scale bars, 10 μm). **H**, RT-PCR of cTTN1 after fractionation of nuclear and cytoplasmic RNA of hiPSC-CMs (n=3). Error bars indicate SEM. *P<0.05; ***P<0.001.

In addition, selective loss of cTTN1 caused gross abnormalities in hiPSC-CMs, where it disrupted sarcomere structure and induced cell death (Figure 2A–2C and Figure VI in the Data Supplement). Furthermore, contraction analysis of engineered heart tissues after selective loss of cTTN1 demonstrated markedly reduced contraction amplitudes 7 and 14 days after generation of the engineered heart tissues (Figure 2D and Figure VII and Movies I and II in the Data Supplement). These contraction amplitudes obtained by the MUSCLEMOTION algorithm highly correlate with absolute force.^25^ However, despite these severe derangements, expression of *NPPA* and *NPPB* was not increased after loss of cTTN1 (Figure 2E), which demonstrates that loss of cTTN1 did not induce a general stress response in these cardiomyocytes. This was further confirmed by RNAseq, which showed that muscle contraction was the only affected pathway in common by both the differentially expressed and differentially spliced genes (Figure 2F and Excel File IV in the Data Supplement).

We further showed by fluorescent in situ hybridization and cell fractionation experiments that cTTN1 localizes to both the nucleus and cytoplasm. This broad distribution, combined with its very large predicted size, indicates that cTTN1 might have different functions according to its location (Figure 2G and 2H). It is conceivable that within the cytoplasm cTTN1 acts by sponging of miRNAs, as has been shown for other circRNAs.^4,5^ Indeed, bioinformatic prediction of miRNA binding sites using CircRNAprofiler^15^ (Excel File V in the Data Supplement) showed ample putative miRNA binding sites, and this was confirmed by finding 6 miRNAs upregulated after inhibition of cTTN1 in hiPSC-CMs (Figure VIIIa and VIIIb in the Data Supplement). We then compared our RNAseq after loss of cTTN1 (Figure 2F) with our RNAseq of RBM20 KO mice,^14^ and our attention was drawn to *MYBPHL*, which was downregulated after loss of cTTN1 and, although with high variability, its expression was reduced in RBM20 KO mice. Furthermore, *MYBPHL* was previously shown to induce a form of DCM in human and mouse.^26^ *MYBPHL* is a predicted target of 3 of the upregulated miRNAs, and its expression is reduced both after inhibition of cTTN1 in hiPSC-CMs and in *RBM20* mutation carriers (Excel File VI and Figure VIIIc–VIIIe in the Data Supplement). Together, these findings indicate that downregulation of *MYBPHL* could also affect the observed phenotype. However, inhibition of miR-34a-5p, the miRNA with 8 binding sites in the *MYBPHL* 3′ untranslated region, in hiPSC-CMs did not relieve *MYBPHL* repression (Figure VIIIf in the Data Supplement).

### cTTN1 Is Necessary for RBM20 Splicing

**Figure 3.**
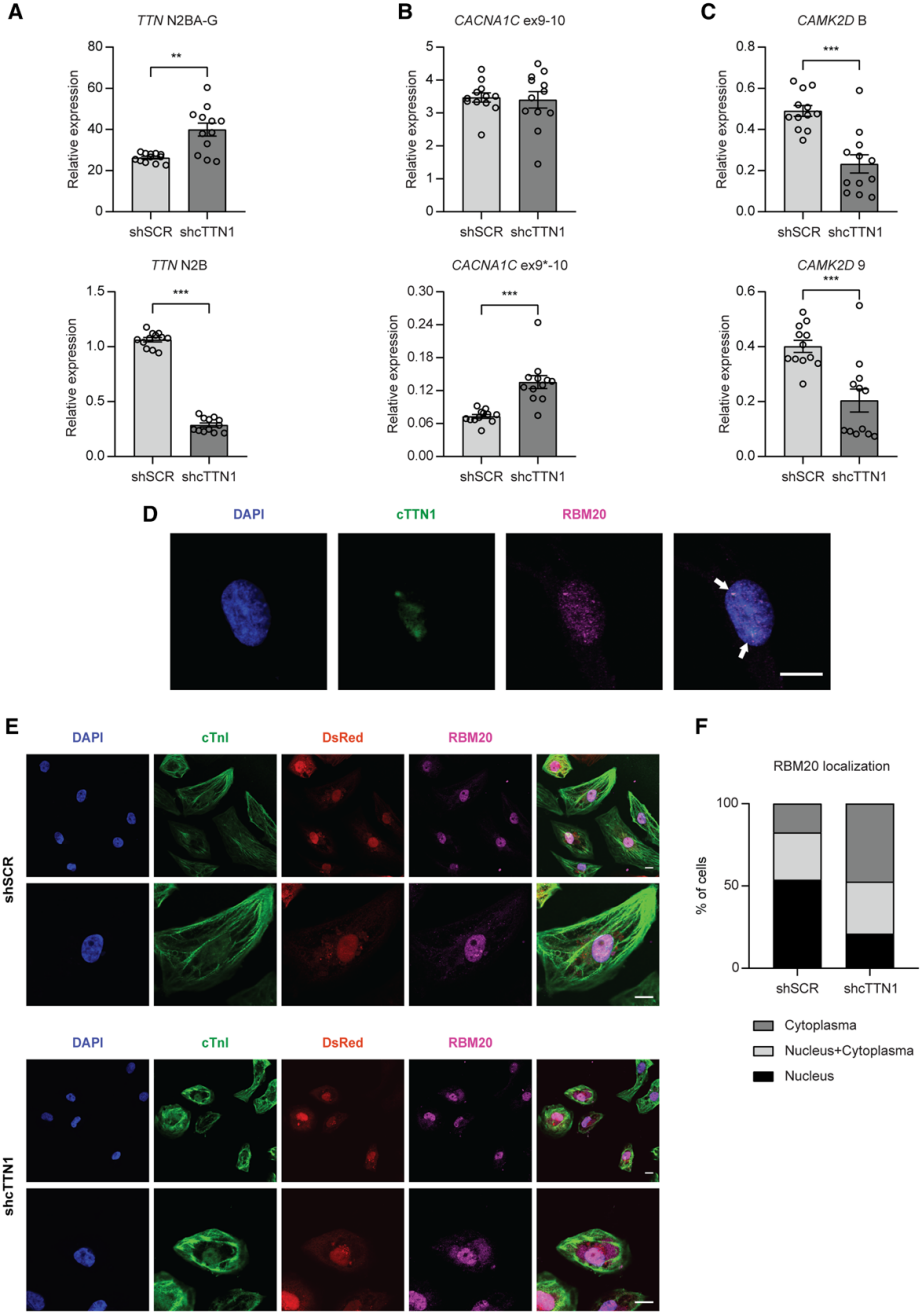
**Loss of cTTN1 leads to reduced RBM20 function and RBM20 mislocalization.** **A–C**, Quantitative reverse transcriptase–polymerase chain reaction of the RBM20 splicing targets *TTN*, *CACNA1C*, and *CAMK2D* after loss of cTTN1 in human induced pluripotent stem cell–derived cardiomyocytes (hiPSC-CMs; n=12; 4 differentiations; **P<0.025; ***P<0.001; error bars indicate SEM). **D**, Representative image of immunofluorescent in situ hybridization for RBM20 (magenta) and cTTN1 (green) in hiPSC-CMs (DAPI, blue). Arrows indicate foci where RBM20 and cTTN1 overlap; scale bars, 10 μm. **E**, Representative pictures at 2 magnifications of immunocytochemistry after loss of cTTN1 in hiPSC-CMs (DAPI, blue; cardiac troponin I, green; dsRED, red; RBM20, magenta; scale bars, 10 μm). **F**, Quantification of **E** for RBM20 localization. N=143 negative control shRNA (shSCR) and 91 shcTTN1 from 4 differentiations. *P*<0.001, χ^2^ test.

We showed earlier that cTTN1 is an RBM20-dependent circRNA^9^ and there already hypothesized that the loss of these RBM20-dependent circRNAs might play a role in the adverse effects caused by loss of RBM20. Indeed, even the selective loss of cTTN1 recapitulates not only the structural derangements seen after loss of RBM20^27^ but also the splice abnormalities caused by loss of RBM20 (Figure 3A–3C and Figure IXa in the Data Supplement). Strikingly, this also involves missplicing of *TTN* itself, which shows that cTTN1 is a crucial regulator of *TTN* splicing, the transcript from which it derives.

Because cTTN1 is formed only in the presence of RBM20 and cTTN1 seems to also regulate the same splicing effects, we asked how cTTN1 is involved in the proper function of RBM20. Loss of cTTN1 does not decrease the levels of *RBM20* itself (Figure IXb in the Data Supplement). However, we find by immunofluorescent in situ hybridization that cTTN1 colocalizes with RBM20 in distinct nuclear foci (Figure 3D). These nuclear RBM20 foci have been shown to be the site where RBM20 exerts its splicing function.^28^ On selective loss of cTTN1, the normal nuclear localization of RBM20 is lost, and RBM20 is translocated to the cytoplasm (Figure 3E and 3F and Figure IXc in the Data Supplement). Together, these results suggest a role for cTTN1 in keeping RBM20 localized to the nucleus to perform its splicing function.

**Figure 4.**
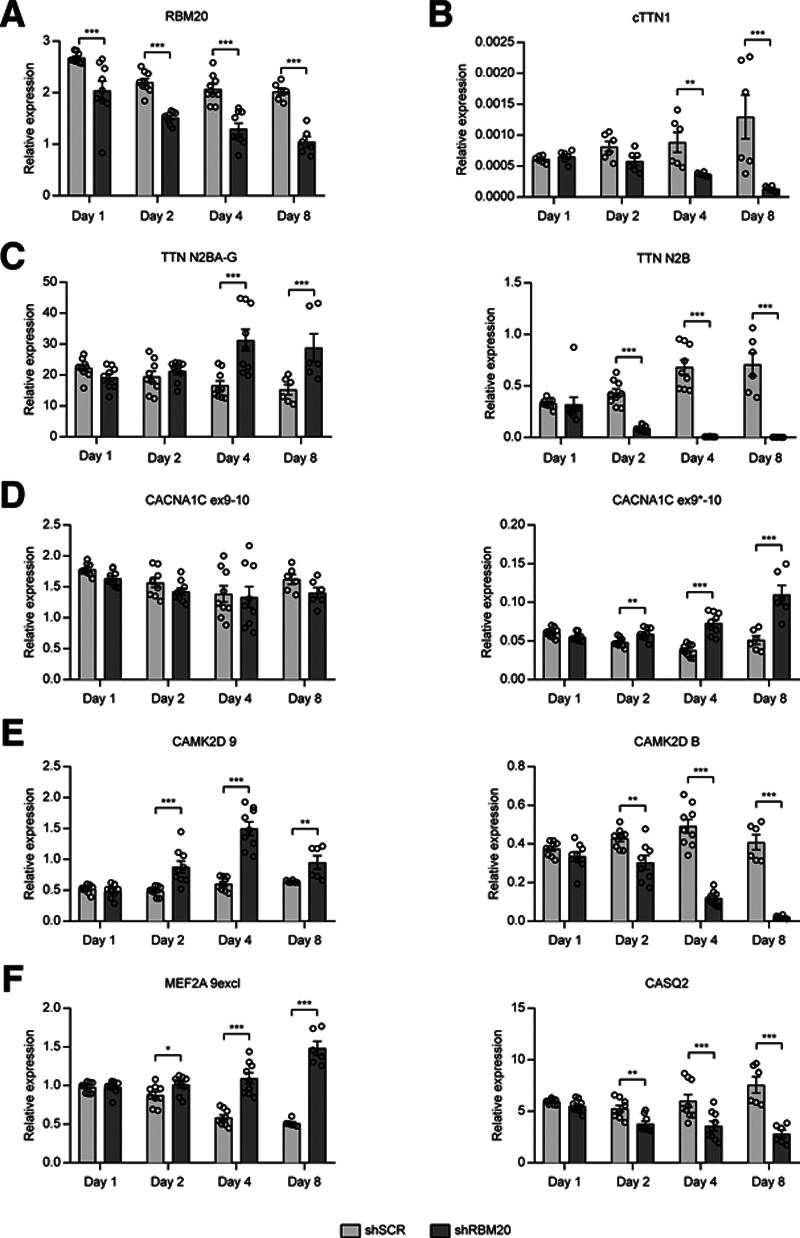
**Time-dependent loss of RBM20 disentangles the RBM20 and cTTN1 effects.** Quantitative reverse transcriptase–polymerase chain reaction after 1, 2, 4, and 8 days of *RBM20* loss in hiPSC-CMs for *RBM20* (**A**); cTTN1 (**B**); the RBM20 splicing targets *TTN* (**C**), *CACNA1C* (**D**), and *CAMK2D* (**E**); and the SRSF10 targets *MEF2A* and *CASQ* (**F**); n=9; 3 differentiations except for day 8 and cTTN1 for every timepoint (n=6; 2 differentiations). Error bars indicate SEM. *P<0.05; **P<0.025; ***P<0.001.

To further disentangle the effects of loss of RBM20 from those of loss of cTTN1, we took advantage of the longer half-life of a circRNA compared with mRNA.^29^ We speculated that after loss of RBM20 by shRNA inhibition, it would take longer for cTTN1 to also disappear. We would thus create a situation in which cTTN1 is still present while RBM20 is already absent. Indeed, on knock-down of *RBM20* in hiPSC-CMs, *RBM20* itself is already lost 1 day after transduction, whereas cTTN1 starts to diminish only from day 2 onward (Figure 4A and 4B). This difference at day 1 allowed us to ask which targets are misspliced when only *RBM20* is lost while cTTN1 is still present. This demonstrated that only the loss of *TTN-N2B* is exclusively dependent on RBM20, whereas the missplicing and regulation of all other RBM20 and SRSF10 targets occurred when cTTN1 also was lost (Figure 4C–4F). This suggests that the missplicing caused by loss of RBM20 is mediated mainly by the subsequent loss of cTTN1. Specifically, the expression of the *CAMK2D-9* isoform, an RBM20 splicing target, is, as expected, upregulated after loss of RBM20, but this upregulation decreases again when cTTN1 is strongly reduced at day 8, which is consistent with the observed downregulation of this *CAMK2D-9* isoform after downregulation of cTTN1 (Figures 3C and 4E). This suggests a negative feedback loop on this specific isoform by loss of cTTN1 after RBM20 loss.

## Discussion

Here, we demonstrate that the *TTN* transcript gives rise to a group of RBM20-dependent circular RNAs that by circularization form a new RNAseq in their back-splice junction that enables them to bind the splice regulator SRSF10 and to regulate its function. The motif thus formed is suggested to be under reduced genetic constraint in the human population, which underlines its biological importance. We show that the highest expressed circRNA within this group, cTTN1, has multiple functions and plays an important role in cardiac homeostasis. Indeed, besides its binding of SRSF10, cTTN1 also colocalizes with the muscle-specific splice regulator RBM20, and we find that it is necessary for proper localization of RBM20 and thereby for RBM20 to splice crucial muscle genes, including the *TTN* transcript itself.

We studied cTTN1 because it is the highest expressed circRNA in the human heart and is downregulated in DCM.^9,10^ The fact that we decided to study a human-specific circRNA has advantages and disadvantages. A strong advantage is that we studied a mechanism immediately applicable to humans. However, this also means we had to study this circRNA in hiPSC-CMs instead of (rodent) primary cells or in vivo. HiPSC-CMs are known to be immature fetal-like cardiomyocytes^30^ with a higher expression of the fetal *TTN* isoform, which includes the I-band region in the transcript. This limits the RNA template for circRNA production of circRNAs derived from the I-band region. As a consequence, cTTN1 had relatively lower expression in these cells compared with the human heart. This suggests that cTTN1 has a more important role in the adult heart and that loss of cTTN1 in the adult heart would result in a more tremendous phenotype.

One could argue that the fact that cTTN1 is not conserved to rodents implies that the mechanisms we describe are less important. However, scrutinizing our previously published RNAseq data of RBM20 KO mice^31^ revealed that the formation of the SRSF10 binding motif in the back-splice junction of RBM20-dependent circRNAs is conserved to mice. The difference between mouse and human is the exons used for back-splicing to create this motif and thereby the exact composition of the full circRNA sequence. The conservation of the motif formation, together with the genetic constraint of the motif in human circRNAs, implies that this mechanism is of biological importance.

We describe that cTTN1 is potentially a very large circRNA that could span 35 to 58 exons (6.6–10 kb). This prediction is based on our analysis using the circAST tool.^24^ This tool reports which exons within a certain region are detected in RNAseq data. We investigated the region from which cTTN1 is derived (*TTN* exons 79–145) in RNAseR-treated RNAseq data, in which all noncircRNAs are degraded before sequencing. This means that all reported exons within this region are potentially derived from cTTN1. However, cTTN1 is derived from the I-band region of *TTN*, from which the majority of circRNAs within *TTN* are derived.^9^ As a consequence, the reported exons in the circAST analysis could also be derived from other circRNAs stemming from this region. In addition, the exons detected in the RNAseq data showed variability between the 3 RNAseq samples that we analyzed (Excel File III in the Data Supplement), which indicates variability in which exons are included in the circRNAs. This could be caused by differences in back-splicing and thus which circRNAs are formed, but it could also be caused by alternative linear splicing that occurred within the formed circRNAs. We defined cTTN1 as the circRNA formed by back-splicing of exon 79 and 145, but based on the circAST analysis, we cannot exclude that several isoforms of cTTN1 exist. This implies that different cTTN1 isoforms could underlie the different mechanisms we describe. Unfortunately, the possible large size of cTTN1 and the uncertainty about its composition mean that overexpression as a possible rescue for RBM20 mutation carriers would be a very uncertain path to take.

RBM20 has been shown to exert its splicing function in nuclear foci.^28^ In these foci, the mRNA of the RBM20 splicing targets is produced from their respective DNA templates and immediately spliced by RBM20.^28^ It has also been shown that most RBM20 mutations occur in the RSRSP domain,^32^ which is necessary for nuclear localization of RBM20. Therefore, *RBM20* mutations in this domain lead to translocation of RBM20 out of the nucleus and, not surprisingly, to defects in splicing of the RBM20 targets.^33^ Furthermore, in homozygous genome-edited pigs with a mutation in this RSRSP domain, ribonucleoprotein granules accumulated abnormally in the cytoplasm.^34^ These dysregulated granules were linked to myocardial cellular pathobiology and heart failure and therefore were introduced as a radical new concept underlying DCM in *RBM20* mutation carriers.^34^ We show that selective loss of cTTN1 also leads to mislocalization of RBM20 from the nucleus to cytoplasm, which thus could lead to the observed phenotype via the loss of splicing in the nuclear foci but probably also via dysregulation of ribonucleoprotein granules.

The experiment in which we detected splicing of RBM20 and SRSF10 targets at several time points after inhibition of RBM20 suggests that cTTN1 affects splicing directly, because in this experiment splicing of these targets is largely preserved as long as cTTN1 is still present while RBM20 is already absent. We therefore hypothesize that cTTN1 is able to recruit other splice factors to the nuclear foci where normally RBM20 is present and exerting its function^28^ and that these factors are able to splice mRNA targets at similar positions as RBM20. This hypothesis is supported by the 4-nt long recognition motif of RBM20 (UCUU),^35,36^ which is also part of the recognition motif of other splice factors. Some of these splice factors are, according to our RNA-binding protein analysis using circProfiler (Excel File II in the Data Supplement), also able to bind cTTN1 in the full circRNA sequence. This would mean that these splice factors splice the same mRNA targets in the nuclear foci as long as cTTN1 is present and recruiting them to these foci.

We find a novel role for the *TTN* transcript in that it produces important regulatory RNA molecules, of which one eventually also regulates splicing of the *TTN* transcript. These circRNAs stem from the I-band region of the *TTN* transcript that hitherto received less attention because it is spliced out in the healthy heart. However, these data suggest that this region is an important source of muscle-specific regulatory RNAs and could potentially also be a source of disease-causing mutations. Therefore, we believe that variants in the I-band region should also be considered when patients are checked for disease-causing mutations. This is even more important when the phenotype resembles the phenotype of DCM with arrhythmias as seen in *RBM20* mutation carriers, whereas no *RBM20* mutation is detected.

### Conclusions

This study provides a first example of a functional RNAsequence formed by circularization that cannot be readily recognized in the human genome and illustrates how circularized RNA contributes to sequence diversity in a functional manner.

## Acknowledgments

The authors thank Malou van den Boogaard, Dylan K. de Vries, Guillermo Griffith, and Corrie de Gier-de Vries for experimental assistance; Esther E. Creemers for scientific discussions; and Cris dos Remedios, Albert Suurmeijer, Marijke Wasielewski, Jan Jongbloed, Jolanda van der Velden, and Torsten Rasmussen for human tissue samples.

## Sources of Funding

This work was supported by grants from ZonMw to Dr Tijsen (Veni 91616150), the Netherlands Cardiovascular Research Initiative to Dr Pinto (CVON-ARENA-PRIME), the Wellcome Trust (107469/Z/15/Z), Medical Research Council (UK), British Heart Foundation (RE/18/4/34215), and the NIHR Imperial College Biomedical Research Centre to Dr Ware.

## Disclosures

None.

## Supplemental Materials

Expanded Methods

Data Supplement Figures I–IX

Data Supplement Tables I–III

Data Supplement Movies I–II

Data Supplement Excel Files I–VI

References 37–44

## Supplementary Material


