# Supplementary material for: Titin Circular RNAs Create a Back-Splice Motif Essential for SRSF10 Splicing
Source: Circulation. 2021 Feb 15;143(15):1502–12. doi: 10.1161/CIRCULATIONAHA.120.050455 (PMC8032209; doi:10.1161/CIRCULATIONAHA.120.050455)
Supplement: Supplementary file 1 [file cir-143-1502-s001.pdf]

SUPPLEMENTAL MATERIAL

Supplemental Methods

Human iPSC culture

The fully characterized hiPSC line derived from a healthy male was previously published.[16] The dermal fibroblast were obtained with informed consent after approval by the IRB committee of Rambam Medical Center. Colonies of these hiPSCs were cultured in mTeSR-1 (StemCell Technologies; 85850) on plates coated with 1:200 diluted growth factor-reduced Matrigel (Corning; FAL356231). Cells were passaged every 4-6 days via dissociation with 0.5 mM EDTA (Invitrogen; 15575-038), and replated in mTeSR-1 supplemented with 2 μM Thiazovivin (Selleck Chemicals; S1459). Between passages mTeSR-1 medium was replaced every day, except for the first day after passaging.

Cardiac differentiation of hiPSC

Cardiac differentiation was performed following a previously published protocol with slight adaptations.[17] Differentiation was induced 3-5 days after passaging by changing to CDM3 medium (RPMI-1640, Gibco 21875; 500 μg/ml human serum albumin, Sigma A9731; 213 μg/ml L-ascorbic acid 2-phosphate, Sigma A8960; 1% penicillin/streptomycin) supplemented with 6 μM CHIR99021 (Stemgent; 04-0004-10) for two days, followed by CDM3 with 2 μM Wnt-C59 (Selleck Chemicals; S7037) for two days. From day 4 to day 10 medium was changed every other day for RPMI/B27 medium (RPMI-1640; 2% B27 supplement minus insulin, Gibco A1895601; 1% penicillin/streptomycin). Spontaneous contraction could be identified from day 8 onwards.

From day 10 onwards medium on hiPSC-CM was replaced once every week with CDM3 medium without glucose (RPMI-1640 without glucose, Gibco 11879) supplemented with 20 mM sodium-lactate (Sigma-Aldrich; L7022) dissolved in 1 M HEPES-solution for at least 2 weeks to metabolically select the cardiomyocytes.[18] After selection medium was replaced once a week with CDM3 medium with glucose.

Purified populations of cardiomyocytes were either dissociated by TrypLE Express (Gibco; 12604) incubation for 15 minutes and plated on matrigel coated plates or coverslips in RPMI/B27 with 2 μM Thiazovivin for further experiments or harvested directly for RNA isolations. All experiments were conducted on hiPSC-CM 40-60 days after start of differentiation and each observation was replicated in 2 to 5 independent experiments with hiPSC-CMs from different differentiations.

Human samples

Control left ventricular tissue samples were derived from hearts, without history of cardiac abnormalities, not used for transplantation due to logistic reasons. Left ventricular tissue from patients with end-stage idiopathic dilated cardiomyopathy (DCM) was obtained from the Sydney Heart Bank and was gathered during heart transplantation surgery with approval of the St Vincent's Hospital Human Research Ethics committee (File number H03/118) and the University of Sydney (HREC number 12146). Left ventricular tissue of the DCM patients with a heterozygous *RBM20* mutation was also obtained during heart transplantation surgery. The sample with the E913K mutation was obtained from a 19-year old male patient at Aarhus University Hospital, Denmark. This mutation is classified as pathogenic and co-segregated within the family. No other mutations were identified in the patient among 83 tested DCM genes.[37] The sample with the R634W mutation was obtained at University Medical Center Groningen, the Netherlands from a 63-year old male. This mutation is classified as pathogenic and segregated within the family. Besides the *RBM20* R634W mutation a *DMD* W1670C mutation was identified in this patient, which was classified as benign/uncertain significance. Our investigation is in accordance with the Declaration of Helsinki and institutional guidelines and all tissue samples are taken after informed consent of the patients.

Plasmid generation

For shRNA expression we used the pLKO.1 backbone either with puromycin as selection marker (pLKO.1-puro; Addgene 8453) or with dsRED as fluorescent marker. To create the PLKO.1-dsRED plasmid, the dsRED coding sequence was amplified from the pIRES2-dsRED-express vector (Clontech; 632463) using the primers in Supplemental Table I. The puromycin resistance cassette was removed from pLKO.1-puro by BamH1 and Kpn1 restriction and the dsRED PCR products were ligated into these restriction overhangs. Cloning of shRNA sequences was similar in the pLKO.1-puro and pLKO.1-dsRED plasmids. Therefore, we designed the following oligonucleotides: forward 5'-CCGGAA-19 bp sense strand-TCAAGAC-19bp antisense strand-TTTTTTG-3' and reverse 5'-AATTCAAAAA AA-19bp sense strand-GTCTTGA-19bp antisense strand-TT-3'. Where the sense strand is exactly the mRNA targeting sequence and the antisense strand its reverse complementary sequence that will eventually bind the mRNA. We annealed 1 nmol of these oligonucleotides and cloned them into the AgeI and EcoRI restriction sites in the pLKO.1 plasmids. ShRNAs targeting the circRNAs were cloned into the pLKO.1-dsRED plasmid and shRNAs targeting

RBM20 and *SRSF10* into the pLKO.1-puro plasmid. ShRNA sequences are detailed in Supplemental Table II.

To create the pCDH-cTTN1 construct, we amplified two amplicons from genomic DNA of hiPSCs (primers in Supplemental Table I): 1) from the middle of exon 78, including intron 78 and exon 79, till the end of intron 79; 2) from the beginning of exon 145, including intron 145, till the middle of exon 146. In a second PCR step we combined these two amplicons and ligated this product into the BamHI and NotI sites of the pCDH-CMV-MCS-EF1-Puro plasmid. We created 2 mutations within the SRSF10 motif of pCDH-cTTN1 (from AAAGAACC to AAGGAGCC) by PCR based mutagenesis. Therefore, we amplified 3 PCR products from the pCDH-cTTN1 plasmid with primers introducing these mutations (Supplemental Table I): 1) from the middle of exon 78 to the introduced mutation in exon 79; 2) from the mutation in exon 79 to the introduced mutation in exon 145; 3) from the mutation in exon 145 to the middle of exon 146. In a second PCR step we combined these amplicons and ligated this product into the pCDH-CMV-MCS-EF1-Puro using the BamHI and NotI sites.

We created a full-length SRSF10 overexpression construct (pCDH-flag-SRSF10) with a N-terminal flag-tag by amplifying *SRSF10* from cDNA derived from the hiPSC-CMs (primers Supplemental Table I). Furthermore, we created a pCDH-flag-EGFP control construct by amplifying EGFP from the pIRES2-EGFP plasmid. Both PCR products were cloned into the BamHI and NotI restriction sites of pCDH-CMV-MCS-EF1-Puro.

All plasmid sequences were verified by Sanger sequencing and occurrence of mutations excluded.

Virus production

To produce third-generation lentivirus of pLKO.1-puro, pLKO.1-dsRED or pCDH-CMV-MCS-EF1-Puro based constructs we co-transfected $4*10^6$ HEK293T cells with 4 µg of the expression plasmid, 2.7 µg pMDLg/pRRE, 1 µg pRSV-Rev, 1.4 µg pVSVG using Genejammer (Agilent; 204130) according to the manufacturer's protocol. The next day medium was replaced to RPMI/B27 for the circRNA targeting viruses and to CDM3 medium for all other viruses. This medium containing the produced lentivirus was collected after 24 hours and either used directly for hiPSC-CM transduction or the amount of transducing units was first determined.

The amount of transducing units is determined by transducing 250.000 HEK293T cells with series of 50/100/200/500/1000 µl of medium with virus of the pLKO.1-dsRED plasmid. Three days after, the cells were trypsinized and FACS sorted for the dsRED positive

population. The condition with 10-20% positive cells was used to calculate the amount of transducing units assuming 1 viral copy per cell. The amount of tranducing units for an experimental virus and its corresponding control were determined in the same FACS experiment.

HiPSC-CM infection

HiPSC-CM were dissociated and replated 2 to 4 days before lentiviral transduction to ensure homogenous cell populations between conditions. Of all viruses containing a puromycin resistance cassette, 2 ml/well of medium with virus was freshly added to the hiPSC-CM in a 6-well plate. After 5 days virus was removed and puromycin selection started with 8 µg/ml puromycin for 72 hours after which the cells were fixed or harvested. These experiments where either completely performed in RPMI/B27 or CDM3 medium depending on the medium the virus was generated in.

In the experiments to select the best shRNA to inhibit the circRNA, medium containing the shRNA-encoding lentivirus was freshly added to the hiPSC-CM and the experiment performed as described above without puromycin selection. In the follow-up experiments using cTTN1-shRNAs the amount of transducing units was the same for the cTTN1-shRNA and the negative control shRNA. The amount of transducing units ranged between 250000 and 850000 per well in 6-well plates for RNA, between 20000 and 120000 per well in 24-well plates for immunocytochemistry, and 15000 per well in 96-wellplates for apoptosis experiments, depending on the density of the plated hiPSC-CMs.

HiPSC-CM transfection

HiPSC-CM were dissociated and replated in 6-wellplates 2 days before transfection to ensure homogenous cell populations between conditions. These cells were transfected with 100 nmol/l miRCURY LNA miRNA power inhibitors (Qiagen; negative control A, 339136 YI00199006-DDA or hsa-miR-34a-5p, 339131 YI04100982-DDA) using 4 µl transIT-LT1 (Mirus; MIR 2304) according to the manufacturer’s protocol. After 48 hours cells were harvested for RNA isolations.

Cytoplasmic/Nuclear fractionation for RNA isolation

10^7 dissociated cells were spinned for 5 minutes at 160x g and gently resuspended in 550 µl precooled membrane lysis buffer (50 mM TRIS pH8.0; 140 mM NaCl; 1.5 mM $MgCl_2$; 0.5% NP-40; 10 mM EDTA; 1 mM DTT; 1 µl/ml Superase-In, Invitrogen AM2696). After 5 minutes

incubation on ice, lysed cells are centrifuged 5 minutes at 500x g at 4°C. Supernatant is transferred to a new tube and recentrifuged to remove any remaining nuclei. The cleared supernatant is transferred to a new tube and 1 ml TriReagent added to start RNA isolation. Nuclei pellet of the first centrifugation is washed in 175 µl precooled membrane lysis buffer, by gentle resuspension and centrifugation. The nuclei pellet is again resuspended in 175 µl precooled membrane lysis buffer, 1 ml TriReagent added, samples are vortexed and 5 times passed through a 20-gauge needle to break the nuclear membrane.

RNA isolation

Total RNA was isolated from all samples using 1 ml TriReagent (Sigma Aldrich; T9424). TriReagent was either added to frozen tissue samples, which were homogenized using a MagNA Lyser (Roche), to a frozen cell pellet after TrypLE Express dissociation, or directly to live cells growing on a dish. Total RNA isolation was performed according to the manufacturer's protocol.

RNA-sequencing & analysis

We performed RNA-seq on 3 biological replicates of hiPSC-CMs transduced with a cTTN1 or negative control shRNA. Therefore, RNA was confirmed to have a RIN score >8 with the Agilent 2100 Bioanalyser. Total RNA samples were sequenced on a Illumina NextSeq 500 platform in paired-end mode with a read length of 150 bp. Sequencing depth was approximately 50 million raw reads per sample. Base-calling was performed using the bcl2fastq 2.0 conversion software from Illumina.

Quality control of fastq files was performed using FASTQC (https://www.bioinformatics. babraham.ac.uk/projects/fastqc/). Trimmomatic version 0.351[38] was used to remove Illumina adapters and low quality bases, using a Phred score cutoff of 30 while discarding reads with a length below 25 bases. The RNA-seq reads were then aligned against the human genome using TopHat2 version 2.0.14[39] with default values and the UCSC hg19 annotation file. Differential gene expression analysis was performed using the R Bioconductor package, DESeq2.[40] GenomicRanges infrastructure was used to count the number of aligned reads overlapping with each gene. To test for exon usage differences we used the R Bioconductor package DEXSeq version 1.20.0[41], using GenomicRanges infrastructure to count the number of aligned reads overlapping with each exonic region (see Supplemental Excel File IV).

Pathway enrichment analysis was performed using the R package ReactomePA. It implements hypergeometric models to assess whether the number of selected genes associated

with a reactome pathway is larger than expected. The gene names of the differentially spliced exons or differentially expressed genes (p-adj cut-off ≤0.05 and absolute log2 fold change ≥1) were used as input for the pathway enrichment analysis.

qRT-PCR for mRNA

To detect mRNA or splice isoform levels, 250 ng to 1 µg RNA was DNAse treated with DNAseI amplification grade (Invitrogen; 18068015) and reverse transcribed using Superscript II reverse transcriptase (Invitrogen; 18064014) with oligo-dT and random hexamer primers according to the manufacturer's protocol. cDNA was diluted 5 times and 2 µl used as input for the qPCR.

qPCR was performed using 1 µM primers (Supplemental Table I) and LightCycler 480 SYBR Green master 1 (Roche; 04887352001) on a LightCycler 480 system II (Roche) using the following cycling program: 5 minutes pre-incubation at 95°C; 40 cycles of 10 seconds denaturation at 95°C, 20 seconds annealing (temperatures in Supplemental Table I), and 20 seconds elongation at 72°C. Data were analyzed using LinRegPCR quantitative PCR analysis software[42] and the starting concentration of transcripts estimated by this software was corrected for the geometric mean of the estimated starting concentration of the three housekeeping genes *HPRT*, *GAPDH* and *TBP*.

(q)RT-PCR for circRNAs

For some circRNA experiments the RNA was first treated with RNase R to remove linear RNA. Therefore we treated 1 µg total RNA with 5 units of RNase R (Epicentre; RNR07250) at 37°C for 10 minutes followed by heat inactivation at 95°C for 3 minutes. Control samples in these experiments were treated similarly without addition of RNase R.

For RT-PCR detection of circRNAs, RNA was DNAse treated and reverse transcribed as described above using a combination of random hexamer primers and oligo dT. For previously RNase R treated samples reaction volumes were doubled. cDNA was diluted 5 times and 1 to 4 µl was used for input in the end-point PCR reactions and 2 µl for input in qPCR.

End-point PCR was performed to detect circRNAs using HOT FIREPol DNA polymerase and 1 µM primers as detailed in Supplemental Table I. Primers are designed in a divergent manner in the two exons that backsplice to create the circRNA of interest. Therefore, the band of interest is the smallest band detected in the agarose gel, which is verified by Sanger sequencing. The program used to detect the circRNAs contained the following cycling conditions: 30 seconds at 95°C denaturation, 30 seconds at variable temperatures

(Supplemental Table I) annealing and 10 seconds at 72°C elongation. The amount of cycli ranged between 30 and 45 depending on the type of experiment, amount of starting material and efficiency of the primers/amount of extra amplified amplicons by the primers.

To quantitatively detect cTTN1 we designed a custom made Taqman primer probe set, which contained a non-modified forward primer 5'-AGAGGTGCCCAAGAAGCTC-3' and reverse primer 5'-ATGGATTCTCCCTGCTTTGC-3' and a FAM-labeled MGB probe 5'-AC CCAAAGAACCTCCAAA-3'. Here the probe was designed on the backsplice junction in such a way that the amount of mismatches between cTTN1 and all other circRNAs containing the SRSF10 motif in their backsplice junction was at least one and for most of them 2-4. We performed real-time PCR using 900 nM primers, 250 nM probe and LightCycler 480 probes master (Roche; 04707494001) on a LightCycler 480 system II (Roche) using the following cycling program: 10 minutes pre-incubation at 95°C; 45 cycles of 15 seconds denaturation at 95°C, 1 minute annealing and extension at 60°C. Data were analyzed using LinRegPCR quantitative PCR analysis software.[42]

qRT-PCR for miRNAs

To detect miRNA levels, 500 ng to 1 μg RNA was DNAse treated and reverse transcribed using the miScript reverse transcription kit (Qiagen; 218161) with the miScript HiFlex Buffer. cDNA was diluted 8 times and 2 μl was used as input for the qPCR.

qPCR was performed using High Resolution Melting Master (Roche; 04909631001) in a reaction where an end-concentration of 2.5 mM $MgCl_2$ and 1 μM primers was used. The forward primer was specific for the miRNA (Supplemental Table I) and the reverse primer complementary to the adapter sequence of the RT-primer in the miScript RT kit was general for all miRNAs. Real-time PCR reactions were performed on a LightCycler 480 system II (Roche) using the following cycling program: 10 minutes pre-incubation at 95°C; 40 cycles of 45 seconds denaturation at 95°C, 45 seconds annealing at 55°C, and 45 seconds elongation at 72°C. Data were analyzed using LinRegPCR quantitative PCR analysis software[42] and the starting concentration of miRNAs estimated by this software was corrected for the geometric mean of the estimated starting concentration of the housekeeping genes *HPRT* and *GAPDH*.

Immunocytochemistry

All cells for immunocytochemistry were plated on 12 mm glass coverslips. Untransduced hiPSC-CMs were cultured for 1 week in RPMI/B27, and transduced hiPSC-CM for 2 weeks as described above, all on matrigel-coated coverslips. Cells were fixed in 4% paraformaldehyde

for 10 minutes at room temperature and washed 3 times in PBS. Cells were permeabilized with 0.1% or 1% Triton X-100 in PBS for 8 minutes and unspecific antibody binding was blocked by 1 hour incubation with 4% goat serum or BSA. Primary antibodies (Supplemental Table III) were diluted in PBS with 4% goat serum or 4% BSA with 1% Triton X-100 and incubated overnight at 4°C or 3 hours at room temperature. Cells were washed 3 times in PBST or PBS and afterwards incubated for 1 hour at room temperature in the dark with 1:250 diluted secondary antibodies (Supplemental Table III) in PBS with 4% goat serum or BSA with 1% Triton X-100. Cells were washed 3 times in PBST or PBS. Nuclei were counterstained with DAPI (1:5000) for 5 min and mounted in Mowiol (Sigma, 81381)

Pictures were taken blinded on a Leica TCS SP8 X/DMI6000 inverted confocal microscope with a 63x oil immersion lens. Quantification of myofibrillar structure was performed by separate scoring for striation and sarcomere organization. For sarcomere organization we used the following scores: 0) No myofibrils; 1) randomly scattered, punctuate or fuzzy appearance of myofibrils; 2) not well organized myofibrils structures with no alignment; 3) slightly organized/aligned myofibrils; 4) high degree of myofibril alignment. For striation we used the following scores: 0) No visible striation; 1) Striation visible in some myofibrils; 2) Clearly visible, distinct striation in almost all myofibrils. Quantification of RBM20 localization was performed by dividing cells in 3 groups: 1) only nuclear; 2) nuclear and cytoplasmic; or 3) only cytoplasmic, and the percentage of cells belonging to each group was calculated.

(Immuno)FISH

Cells were dissociated and replated on matrigel coated 12 mm coverslips. While replating 20000 TU of a cTTN1 shRNA or negative control shRNA virus was added to the RPMI/B27 medium with thiazovivin in which the cells were plated to allow immediate transduction. 6 days after transduction, cells were washed in PBS and fixed in 4% formaldehyde (v/v) and 0.05% (v/v) glacial acid in PBS for 17 minutes at room temperature. After 3 PBS washes of 5 minutes each, ice-cold 70% ethanol is added to the cells and they are incubated for 20 minutes at -20°C. Cells are again washed in PBS and incubated 30 minutes at room temperature in 1% Triton X-100 and 1% Saponin in PBS. After another PBS wash, cells are incubated for 15 minutes at 37°C in proteinase K (4 µg/ml in PBS) in the FISH experiments and 5 to 7.5 minutes depending on cell density for the immunoFISH experiments. Cells are washed again in PBS, followed by post-fixation in 4% formaldehyde and 0.05% glacial acid in PBS for 5 minutes. Cells were then incubated for 30 minutes in 3% peroxide in water and washed in PBS again.

This washing step is followed by 3 steps of 5 minutes each of increasing ethanol concentrations from 70 to 100% ethanol after which cells are air-dried. To detect the cTTN1, cells were incubated overnight at 37°C with 10 ng of a FAM-labeled DNA-probe directed towards the cTTN1 backsplice junction (5'-TAATTTCT[FAM-]TAACAAACTT[FAM-]TGGAGGTTCT T[FAM-]TGGGTGGTGG[FAM-]TGCTTCCACT[FAM]TTTTTC[FAM]-3'; IBA life sciences). The next day the probe was washed away in 3 washes of 10 minutes at 60°C in 2x SSC buffer and 3 washes in 0.2x SSC buffer, followed by a PBS wash. Afterwards cells were blocked in 10% horse serum in PBS for 30 minutes at room temperature. For immunoFISH experiments cells were incubated for 3 hours with an anti-RBM20 antibody (Supplemental Table III) diluted 1:100 in 10% horse serum in PBS, washed in PBS, and incubated for 1 hour with an goat-anti-rabbit-alexa647 antibody 1:250 in horse serum in PBS. For FISH and immunoFISH experiments cells were washed in PBS and incubated for 1 hour with an anti-FITC antibody 1:600 diluted in horse serum in PBS. Cells were washed again in PBS and incubated for 15 minutes with a TSA plus fluorescence kit (Perkin Elmer, NEL741001KT). After another PBS wash, cells were incubated for 5 minutes with 1:1000 DAPI in PBS, washed again in PBS and mounted in prolong glass antifade (Invitrogen; P36982). Pictures were taken on a Leica TCS SP8 X/DMI6000 inverted confocal microscope with a 63x oil immersion lens.

Western blot

Protein was extracted in RIPA buffer (50 mM Tris-HCl pH8; 150 mM NaCl; 1% Igepal CA-630; 0.5% sodium deoxycholate; 0.1% sodium dodecyl sulfate) with addition of a protease inhibitor cocktail (Roche; 4693159001) and 0.5 nM Orthovanadate (Sigma; 56508). The lysate was cleared of cell debris by centrifugation for 10 minutes at 10.000g and 4°C. Protein concentrations were measured using the BCA protein assay kit (Pierce; 23225) according to the manufacturer's protocol.

For the western blots we added a 2x sample buffer (17.5% glycerol; 6% SDS; 250 mM Tris-HCl pH6.7; 10% β-mercapto-ethanol) and denatured the protein for 10 minutes at 95°C. Total protein (20 μg) was separated by electrophoresis on pre-cast 4-20% gradient polyacrylamide gels (Bio-Rad; 4561094) and transferred to a methanol activated PVDF membrane (Bio-Rad; 170-4272) using a Trans-Blot Turbo transfer system (Bio-Rad) for 10 minutes at 25V. This membrane was blocked for 1 hour in 5% non-fat dry milk in TBST and incubated with the first antibody (Supplemental Table III) in 5% non-fat dry milk in TBST overnight at 4°C. The next day membranes were washed 3 times 10 minutes in TBST, incubated 1 hour at room temperature with the HRP-linked secondary antibody 1:5000 in 5%

non-fat dry milk in TBST, and washed 3 times for 10 minutes in TBST. Bands were detected using the ECL prime western blotting detection reagent (Amersham; RPN2236) and images acquired using the ImageQuant LAS4000 (GE Healthcare).

RNA immunoprecipitation

HiPSC-CMs (~20*10^6 per RIP experiment including 2 antibodies) for immunoprecipitation of endogenous SRSF10 were collected by TrypLE Express dissociation. For immunoprecipitation of SRSF10 in HEK293T cells we co-transfected 4 µg pCDH-cTTN1 or pCDH-cTTN1mutant with 4 µg pCDH-flag-SRSF10 or pCDH-flag-EGFP into 4*10ˆ6 HEK293T cells using genejammer according to the manufacturer's protocol and these cells were collected 48 hours after transfection. Cells were lysed using 1 volume lysisbuffer compared to the volume of the cell pellet according to the protocol of the MagnaRIP RNA binding protein immunoprecipitation kit (Millipore; 17-700). Immunoprecipitation was further performed using 100 µl lysate input per antibody according to the manufacturer's protocol or repeated under more stringent criteria by adding 1 or 3 M Urea to the washing buffer and blocking of the beads with 1% ultrapure BSA (Invitrogen; AM2616) in RIP wash buffer for 1 hour before the lysate was added. After immunoprecipitation and RNA isolation according to the protocol, cDNA is prepared and circRNAs are detected as described above.

Apoptosis measurements

Apoptosis was measured using the Caspase-Glo 3/7 Assay (Promega; G8090). Therefore hiPSC-CMs were plated in 96-wellplates and transduced with 15.000 transducing units of cTTN1 or negative control shRNAs. Apoptosis was measured 4 days after transduction according to the manufacturer's protocol on a Glomax multi detection system (Promega).

For the TUNEL staining were hiPSC-CMs transduced with cTTN1 or negative control shRNA while being plated on 12 mm glass coverslips. Cells were fixed in ice-cold methanol for 10 minutes 4 days after transduction. After fixation cells were air-dried at room temperature for 5 minutes and rehydrated for 10 minutes in PBS. Cells were stained in 25 µl of TUNEL reaction mix (In Situ Cell Death Detection Kit; Roche Diagnostics; 11684817910) for 1 hour at 37°C. Nuclei were counterstained with DAPI (1:5000) and cells were mounted in Prolong glass antifade (Invitrogen; P36982). Pictures were taken blinded on a Leica TCS SP8 X/DMI6000 inverted confocal microscope with a 20x oil immersion lens and quantified by calculating the percentage of TUNEL positive cells in 5-7 randomly selected areas per coverslip.

For the Annexin V FACS analysis were hiPSC-CM transduced with cTTN1 or a negative control shRNA virus with a DsRED marker while being plated in 12-well plates. After 4 days cells were dissociated with TrypLE express and stained for 10 minutes with 1.65 µg/ml ATTO-488 conjugated Annexin V antibody (Supplemental Table III) in sterile binding buffer (10 mM HEPES/NaOH, pH 7.4; 140 mM NaCl; 2.5 mM $CaCl_2$). Afterwards, cells were washed twice in binding buffer and analyzed by flow cytometry using a Fortessa Cell Analyzer 5 laser 18 color (Becton Dickinson). The percentage of apoptotic cells was calculated as percentage of Annexin V positive cells within the DsRed positive population using FlowJo 10 software for analysis.

Engineered Heart Tissue generation and contraction analysis

Each EHT contained 1.3-1.5*10^6 hiPSC-CM, transduced with cTTN1 or negative control shRNAs 3 days before EHT generation. Fibrin-based EHTs were generated as described previously.[43] Briefly, casting molds were generated with Teflon spacers (EHT Technologies; C0002) placed in 2% liquid agarose (Millipore;121853) in 24 well-plates. Agarose was allowed to solidify and silicon racks containing 2 posts per well (EHT Technologies; C0001) were positioned in the casting molds. The cell-hydrogel suspension consisting of hiPSC-CMs, fibrinogen, thrombin and matrigel was then poured around the posts and incubated at 37°C in a cell incubator for 1.5-2 hours to enable polymerization. EHTs adhered to the silicon racks were transferred to culture medium (DMEM/F12 low glucose, Sigma Aldrich D5546; 5% heat inactivated horse serum; 1% penicillin/streptomycin; 0.1% (w/v) Aprotinin, Sigma-Aldrich A1153; 0.1% (w/v) Insulin, Sigma Aldrich I9278) and medium was replaced 3 times a week. EHTs demonstrated regular contractions 4-6 days after generation. Video-optical recordings of the EHTs (60 frames per second) were acquired at 7 and 14 days after EHT generation and contraction profiles were assayed using Musclemotion™ algorithm.[25]

Calculation of genetic constraint of genomic regions

We compared the observed number of rare variants (allele frequency<0.1%) in the gnomAD reference population (v2.1.1) exome datasets (125,748 individuals) with the number of variants that we would expect to see under neutral variation. In this comparison, the number of observed variants is the count of unique substitutions within the exome data set.

The number of expected variants under neutral selection can be predicted based on sequence-context and methylation level.[19] Therefore we calibrated the baseline mutation rate

to probabilities of neutral substitutions within the 125,748 exomes in gnomAD based on the baseline per-base, per-generation substitution rate estimated from variants in intergenic or intronic regions by gnomAD.[21] Therefore, the baseline mutation rate was regressed to the probabilities of neutral substitutions in the exome, where the proportion of substitutions was calculated using all possible synonymous variants. To prevent bias by low sequence coverage, we only included sites with a median depth of 40 in this regression. We fitted two linear regression models, for substitutions at CpG and non-CpG sites respectively, where the methylation level of CpG sites was provided by gnomAD (Supplemental Figure IIa). The predicted proportions of substitutions of these regression models allow us to estimate the expected number of single-nucleotide variants under neutral selection for any genomic region in this dataset.

In a second step the probabilities of neutral substitutions were adjusted for low coverage (median depth <40). Therefore, the expected number of variants for a genomic region was calculated using the model in step 1 and a second linear regression model is fitted to predict the Observed/Expected ratios for a given sequencing coverage on a $\log_{10}$ scale (Supplemental Figure IIb). The predicted Observed/Expected ratios from this model are used as correction factors to adjust the expected number of variants at low coverage sites.

Confidence intervals for the Observed/Expected ratio were derived by modeling the number of neutral substitutions as a random variable for which the distribution is derived by 10.000 simulations of Bernoulli trials using the probabilities of substitutions estimated above. This is based on the assumption that under neutral selection it is a Bernoulli trial whether a type of substitution occurs, where the success probability of the trial is the estimated probability of neutral substitution for the corresponding sequence-context of the site. A second assumption is that occurrence of each substitution is independent of each other. This is further clarified by the following example, where we will estimate the number of neutral substitutions at genomic locus chr2:179575882. This locus has A as reference allele and AAA as tri-nucleotide sequence context and is situated in a non-CpG site. There are three possible substitutions (AAA→ACA; AAA→ATA; AAA→AGA) and for each of them the occurrence is a Bernoulli trial (thus $I_{AAA \rightarrow ACA} \sim Bernoulli\ (p_{AAA \rightarrow ACA})$. Under assumption that each substitution is independent, the number of neutral substitutions n at this genomic locus would be the number of successes for the three Bernoulli trials: $n = I_{AAA \rightarrow ACA} + I_{AAA \rightarrow ATA} + I_{AAA \rightarrow AGA}$. Thus for a specific genomic region, the number of neutral substitutions is the sum of Bernoulli random variables corresponding to each possible substitution within the genomic region, for which we can find the distribution through simulations. Here we derived the simulated distribution from 10.000

simulations and used the 5th and 95th percentile points of this distribution to divide the observed number of substitutions with and thereby construct the 90% confidence interval of Observed/Expected.

To validate our model, we applied it to three sets of variants and evaluated their genetic constrains: *TTN* synonymous variants; *TTN* missense variants; *TTN* truncating variants, where synonymous and missense variants are assumed to be under minimal selection, while truncating variants are known to cause DCM. In these analyses the worst molecular consequence of a variant was considered, meaning that the set of truncating variants included nonsense variants and variants that disrupted a canonical splice sequence.

The code used to perform the genetic constraint analysis is available at https://github.com/ImperialCardioGenetics/cTTN. This code was run in R3.6.3 with as required package ggplot2.

Bioinformatic prediction of RBP and miRNA binding

For this prediction we used the raw sequencing data (NCBI accession number PRJNA533243) of our previously published study.[9] In this study we used the paired-end RNA-seq reads of 3 human control hearts, 3 DCM and 3 HCM hearts and aligned them against the human genome reference hg19. We aligned them using MapSplice version 2.2.0 with the settings: min-fusion-distance 200; filtering 1; and min-map-len 25; using CircMarker (release July.24.2018) with the default options and NCLscan v1.4 with the setting span range 25 bases.

CircRNA prediction results were filtered based on read counts (≥5 present in at least 3 samples of 1 condition), which resulted in 1458 circRNAs used for the RBP predictions based on ATtRACT and the miRNA predictions. These predictions are performed using a homemade R-based computational framework, circRNAprofiler (version 1.2.1), which is available on Bioconductor and on github page: https://github.com/Aufiero/circRNAprofiler [15]

RBP predictions were performed on the full predicted circRNA sequences (no alternative splicing assumed) or the backsplice junctions (BSJ) and comparisons were made between 13 RBM20 dependent and 9 RBM20 independent *TTN*-derived circRNAs; 46 *TTN*-derived circRNAs and 1412 circRNAs derived from other genes; cTTN1 and linear *TTN* (hg19 ENST00000589042.1); *TTN* exons possibly included in the above circRNAs with stringent expression criteria (2-49,69-157,207,208,219-240) and *TTN* exons not included in these circRNAs (1,50-68,158-206,209-218,241-363). The target sequences were retrieved using modules 9 and 10 from circRNAprofiler. RBP analysis was performed on these target sequences by using module 12 with the following settings: width = 6, database = ATtRACT,

rbp = TRUE, reverse = FALSE. Normalization was performed by dividing the number of occurrences of each motif by the total length of the circRNA or adjusted BSJ sequences. E.g. by setting width = 6, the BSJ sequences of 22 nucleotides retrieved by using module 10 are trimmed by module 13 so that only 5 nucleotides are left at each side of the BSJ. Only motifs crossing the BSJs with at least one nucleotide are reported. RBM20 motif was included in motifs.txt (as specified by circRNAprofiler) since the motif of this RBP is not reported in ATtRACT database. A custom script with an adapted version of module 9 was used for RBP prediction on linear *TTN* using the same settings. Raw and normalized counts are reported in the Supplemental Excel Files I and II. For the prediction with MEME suite, we used default settings and as input only the circRNAs which were validated by RT-PCR to be RBM20 dependent (Supplemental Figure Ia).

MiRNA binding predictions were performed on the full cTTN1 sequence (no alternative splicing assumed) using module 13 of circRNAprofiler. In the analysis we only included miRNAs expressed in the left ventricle, therefore we used the raw data of a study in which miRNA deep sequencing of the left ventricles was performed under physiological conditions[44] and filtered these data based on an average expression of at least 10 reads in the 4 control samples. This resulted in inclusion of 361 miRNAs in the file miRs.txt as input for circRNAprofiler for the prediction of miRNA binding sites using different stringency cutoffs (Supplemental Excel Files V and VI; sheet 1 totalMatches=7, maxNonCanonicalMatches=0; sheet 2 totalMatches=7, maxNonCanonical Matches=1; sheet 3 totalMatches=6, maxNonCanonical Matches=2).

Bioinformatic prediction of exons included in cTTN1

For this prediction we aligned the paired-end reads of 3 RNAseR treated RNA samples isolated from human left ventricular tissue[12] against the human genome using TopHat2 version 2.0.14[39] with default values and the UCSC hg19 annotation file. We then used the computational tool circAST[24] to determine which exons within the locus of cTTN1 (exon 79-145) are detected in the RNAseR treated samples to determine which exons are derived from circRNAs in this region and could be included in full-length cTTN1 (Supplemental Excel File III).

Statistics of molecular experiments

Data obtained from hiPSC-CM are a combination of 2 to 5 independent experiments on cells from independent differentiations, with at least n=2 biological replicates per independent

experiment. Data of these independent experiments are combined using Factor Correction[20], where the control condition was used as a reference to calculate the correction factor for which all the datapoints of that experiment were corrected. As a consequence shown data for continuous variables are a mean+/-SEM of n=6-15 biological replicates derived from 2-5 differentiations. For categorical data the percentage of cells in all groups is depicted per condition.

To compare continuous variables between two groups we used the Mann-Whitney U-test and between three groups we used the Kruskal-Wallis test combined with the Dunn post-hoc test, these tests were performed in GraphPad Prism Software version 8. To compare the distribution of cells in different categories (e.g. scoring of phenotype) between two groups we used the chi-square test. To compare the effect of loss of RBM20 over time (day 1-8), we made use of a 2-way ANOVA to test the effect of loss of RBM20, the day effect and the interaction term. To fit the linearity condition we performed a ln transformation for cTTN1, *N2BA-G*, *CACNA1 ex9*-10*, and *CAMK2D_9*. In case the interaction term was significant we determined the differences between the shRNA against *RBM20* and the negative control shRNA by pairwise comparison, in which we used Bonferroni correction for multiple testing. Only in *RBM20* and *CACNA1 ex9-10* the interaction term was not significant. In case of *CACNA1 ex9-10* the condition (loss of RBM20) was also not significant in the ANOVA indicating no significant effect of loss of RBM20 and in the case of *RBM20* the condition was significant, indicating a significant effect of the shRNA against *RBM20* at all timepoints, which was further confirmed by the pairwise comparison. The chi-square tests and the analysis of the RBM20 time-points were performed using IBM SPSS statistics version 26. All performed tests were two-sided and a p-value <0.05 was considered significant.

Data and code availability

Raw sequencing data for circRNA detection in human heart tissue are available via NCBI accession number PRJNA5333243 and for cTTN1 inhibition in hiPSC-CM via NCBI accession number PRJNA630157. All analysis output of sequencing data and circRNAprofiler output is included in the Supplemental Excel Files. Data of RNAseR treated human heart tissue RNA sequencing were used under license for the current study and are not publicly available. Data are however available via the corresponding authors upon reasonable request from Stefan Engelhardt. The homemade R-based computational framework, circRNAprofiler is available on Bioconductor and on github page: https://github.com/Aufiero/circRNAprofiler.[15] And the

code for the genetic analysis is available on github page: https://github.com/ImperialCardioGenetics/cTTN.

Supplemental Table I. Primers used for cloning and (q)RT-PCR

Gene	Forward primer (5'-3')	Reverse primer (5'-3')	Annealing temp
**Plasmid generation**			
dsRED	ACTGGGATCCACAACCATGGCCTCCTCC	ACTGGGTACCTAGAGTCGCGGCCGCTAC	55
TTN exon 78 – intron 79	AGCTGGATCCAGGCAAAGGAGACTCTGGTC	GATGAGCTTCTTGGGCACCTCTGGCACTACCAGAAAAAGGAGATAAACAATGAGAAC	58
TTN exon 145 - exon 146	GTTCTCATTGTTTATCTCCTTTTTTCTGGTAGTGCCAGAGGTGCCCAAGAAGCTCATC	CTAGGCGGCCGCTCCTGAGGTAGAGCTACAGG	58
Full insert cTTN1 (combination of PCR products)	AGCTGGATCCAGGCAAAGGAGACTCTGGTC	CTAGGCGGCCGCTCCTGAGGTAGAGCTACAGG	58
TTN exon 78 - exon 79 mutant	AGCTGGATCCAGGCAAAGGAGACTCTGGTC	TCTAATTTCTTAACAAACTTTGGAGGCTCTAGTAAACCAAACAAAAC	58
TTN exon 79 mutant - exon 145 mutant	TGTTTTGTTTGGTTTACTAGAGCCTCCAAAGTTTGTTAAGAAATTAG	TGGCTCTGTTACAGATTAATGTACCCTTGGGTGGTGGTGCTTCCAC	58
TTN exon 145 mutant – exon 146	AGTGGAAGCACCACCACCCAAGGGTACATTAATCTGTAACAGAGCC	CTAGGCGGCCGCTCCTGAGGTAGAGCTACAGG	58
Flag-SRSF10	ACTGGGATCCGCCACCATGGACTACAAAGACGATGACGACAAGTCCCGCTACCTGCGTCCC	ACTGGCGGCCGCTCAGTGGCCACTGGACTTAGG	61
Flag-EGFP	ACTGGGATCCGCCACCATGGACTACAAAGACGATGACGACAAGGTGAGCAAGGGCGAGGAGC	ACTGGCGGCCGCTTACTTGTACAGCTCGTCCATGC	61
**mRNA and splice isoforms detection**			
TTN exon 1-2	TCATGTCGGAGATGGTCAG	TACCCTCCAGTACCACAACG	60
TTN exon 49-50	TCCAATGAGTATGGCAGTGTCAG	TGAAGGTGAGAGTGCAGCAC	60
TTN exon 49-219	TCCAATGAGTATGGCAGTGTCAG	ACTTTCTTTTCACCTCCAGGC	60
TTN exon 78-79	AGGCAAAGGAGACTCTGGTC	GTTCGCTGTCATTTCGGAACC	60
TTN exon 145-146	TGCCAGAGGTGCCCAAGAAG	TCCTGAGGTAGAGCTACAGG	60
STAT1	ATCACATTCACATGGGTGGAGC	ACAGATACTTCAGGGGATTCTCAGG	60
OAS1	CATCCGCCTAGTCAAGCACT	AAGACCGTCCGAAATCCCTGG	60

SRSF10	TGCCCAGGGGGATCGAAAG	AAGACCGACTTCTTGATCTC CTCC	60
RBM20	GCTGGCATCCGGTGTATAC	CTGAGTGAATGACCTTGC	60
CASQ2	TCCTGGAGATCCTGAAACA GGTTG	TCTGTGACATTCACCACCCC AATC	60
GAPDH	ACCCACTCCTCCACCTTTGA C	ACCCTGTTGCTGTAGCCAAA TT	60
HPRT	TGACACTGGCAAAACAATG CA	GGTCCTTTTCACCAGCAAGC T	60
TBP	GCTCACCCACCAACAATTT AG	TCTGCTCTGACTTTAGCACC TG	60
MEF2A exon 8/10-10	GCATGATGCCTCCACTAAA TACCC	AGTACACAAGTCCTTGCGGA G	60
CACNA1C exon 9-10	ATGAGGACGAAGGCATGGA TG	GATGTCACCTCCAGCCACG	60
CACNA1C exon 9*-10	CGGGCATGCTTGATCAGAA G	CCGCAGTTTTCTCCCTCGAT	60
CAMK2D-B	AGAAACCAGATGGAGTAAA GAAAAGG	AGCTTCGATCAGTTGTTCAG TGAC	65
CAMK2D-C	ACCAGATGGAGTAAAGGAG TCA	AGCTTCGATCAGTTGTTCAG TGAC	60
CAMK2D-9	ACCAGATGGAGTAAAGGAG CCC	AGCTTCGATCAGTTGTTCAG TGAC	60
**CircRNA detection**			
cTTN1(145-79)	AAAAGTGGAAGCACCACCA C	ATGGATTCTCCCTGCTTTGC	58
cTTN 1(145-79)	AGAGGTGCCCAAGAAGCTC	GTTCGCTGTCATTTCGGAAC C	58
cTTN 4(122-89)	CCCAGAGGTGCCAAAGAAA C	TGGAGACCCACCGATTTTG	58
cTTN 5(151-89)	AGTTGAAGAGGTGGCACCA C	TGGAGACCCACCGATTTTG	58
cTTN 6(145-143)	AAAAGTGGAAGCACCACCA C	TTCTTGGGTACCTCTGGCAC	58
cTTN 7(152-69)	ACCACCTAGAGTGCCTGAA G	TGAAGAGTCACCTGCTTTCA C	58
cTTN 10(49-2)	AGAACCCAGGTGGTTGACT GC	CGCTTTGTAACGGCTGCGTA AAC	58
cTTN 14(49-48)	AGAACCCAGGTGGTTGACT GC	CCTTCTTCCTCGGCAGACAG	58
cTTN 16(117-116)	GGACTCCAGTACAGGAAG	CCTCCATTCTTCGAGAAGTC	58
cTTN 17(117-89)	GGACTCCAGTACAGGAAG	TGGAGACCCACCGATTTTG	58
cTTN 18(117-79)	GGACTCCAGTACAGGAAG	ATGGATTCTCCCTGCTTTGC	58
cTTN 19(145-135)	AAAAGTGGAAGCACCACCA C	TCCTCATATTCTTCTTCCCG	58

cTTN 21(83-69)	GCCAATGTAGCTGGTTCCG	TGAAGAGTCACCTGCTTTCAC	58
cTTN 22(136-127)	TCCCAGAAGAACCAGTTCC	CTGAAATGGACACACCTTCCTC	58
cTTN 23(157-151)	GTACGTGTTCCTGAAGAGCC	CTTCCTCTATGCTAGGTGGTTC	58
cTTN 24(145-80)	AAAAGTGGAAGCACCACCAC	CAGTGATATTCCCCGACATCGG	58
cTTN 25(157-135)	GTACGTGTTCCTGAAGAGCC	TCCTCATATTCTTCTTCCCG	58
cTTN 26(152-135)	GCAGTACCTGAAGCACCTAC	TCCTCATATTCTTCTTCCCG	58
cTTN 27(240-219)	AAACAGCCACCTTCGACTGC	GCGAACTTTCTTTTCACCTCCAGG	58
cTTN 28(136-135)	AACCAGAGAAGCCTATCC	CTCTTCCATTATAGTTACTTCTT	58
cTTN 29(83-79)	GCCAATGTAGCTGGTTCCG	ATGGATTCTCCCTGCTTTGC	58
cTTN 30(145-88)	AAAAGTGGAAGCACCACCAC	GCGAATCTCTCGGTTATCTTTGGC	58
cTTN 31 (82-78)	GTGTAGTGACTGGAACACCAG	ATCGGTTGAGCACCAGTAAC	58
cTTN 32(82-69)	GTGTAGTGACTGGAACACCAG	TGAAGAGTCACCTGCTTTCAC	58
cTTN 33(157-143)	GTACGTGTTCCTGAAGAGCC	CTTTGGCAGGTGGAGCTTC	58
cTTN 34(145-127)	AAAAGTGGAAGCACCACCAC	CTGAAATGGACACACCTTCCTC	58
cTTN 35(151-82)	AGTTGAAGAGGTGGCACCAC	CTGGTGTTCCAGTCACTACAC	58
cTTN 36(145-134)	AAAAGTGGAAGCACCACCAC	CTTTGATGGGTTGAGGTTCTC	58
cTTN 38(135-89)	AGCTGTTTCAGTACAACGGGAAG	TGGAGACCCACCGATTTTG	58
cTTN 39(157-134)	GTACGTGTTCCTGAAGAGCC	CTTTGATGGGTTGAGGTTCTC	58
cTTN 40(157-89)	GTACGTGTTCCTGAAGAGCC	TGGAGACCCACCGATTTTG	58
cTTN 41(152-89)	GCAGTACCTGAAGCACCTAC	TGGAGACCCACCGATTTTG	58
cTTN 42(152-79)	GCAGTACCTGAAGCACCTAC	ATGGATTCTCCCTGCTTTGC	58
cTTN 43(157-79)	GTACGTGTTCCTGAAGAGCC	ATGGATTCTCCCTGCTTTGC	58
cTTN 44(122-79)	CCCAGAGGTGCCAAAGAAAC	ATGGATTCTCCCTGCTTTGC	58
cTTN 45(120-79)	TGAGCTACCTGAGAAACCAGC	ATGGATTCTCCCTGCTTTGC	58

cTTN 46(145-82)	AAAAGTGGAAGCACCACCAC	CTGGTGTTCCAGTCACTACAC	58
cTTN 47(145-69)	AAAAGTGGAAGCACCACCAC	TGAAGAGTCACCTGCTTTCAC	58
cTTN 48(122-69)	CCCAGAGGTGCCAAAGAAAC	TGAAGAGTCACCTGCTTTCAC	58
cTTN 50(145-81)	AAAAGTGGAAGCACCACCAC	TGCAGAGCATTCTCTCATTCCAG	58
cTTN 51(129-79)	TGACAGAGAGGCAGGAGAAG	ATGGATTCTCCCTGCTTTGC	58
cTTN 52(152-82)	GCAGTACCTGAAGCACCTAC	CTGGTGTTCCAGTCACTACAC	58
cTTN 53(152-78)	GCAGTACCTGAAGCACCTAC	ATCGGTTGAGCACCAGTAAC	58
cTTN 54(157-82)	GTACGTGTTCCTGAAGAGCC	CTGGTGTTCCAGTCACTACAC	58
cTTN 55(145-145)	AAAAGTGGAAGCACCACCAC	GAGCTTCTTGGGCACCTCT	58
cTNNI3K	AATGCAAGGTCCTATGCTGC	TCTGCTTTGATGGTGTACCG	58
cCEP112	AAGTTTCAGATGGAGAAAAGTCATT	TGGTAACATCTTTCAAGTTCTGC	58
cLDLRAD3	GAGCAGAATGCGTCGGAAG	GATGGCATAGGTGATGCTGG	58
cSLC8A1	AAAACCATCGAAGGGACTGCC	ACACTTCCAACTGTCACAACC	58
cDMD-1	GGGAACAGATCCTGGTAAAGC	GATGTTTGCCCATCGATCTCC	58
cCAPZA1	GTCGTTGGAGATCAGAGTGG	CAGGCGTGAACTGATCCATG	58
**MiRNA detection**			
miR-214-3p	ACAGCAGGCACAGACAGGCAGT	GAATCGAGCACCAGTTACGC	55
miR-208a-5p	GAGCTTTTGGCCCGGGTTATAC	GAATCGAGCACCAGTTACGC	55
miR-30b-3p	TGTAAACATCCTACACTCAGCT	GAATCGAGCACCAGTTACGC	55
miR-34a-5p	TGGCAGTGTCTTAGCTGGTTGT	GAATCGAGCACCAGTTACGC	55
miR-320a-3p	AAAAGCTGGGTTGAGAGGGCGA	GAATCGAGCACCAGTTACGC	55
miR-103-5p	AGCTTCTTTACAGTGCTGCCTTG	GAATCGAGCACCAGTTACGC	55

All primers and the annealing temperatures they were used on are included in this table.

Supplemental Table II. shRNA targeting sequences

Gene/CircRNA	shRNA-number	Targeted RNA sequence	Antisense strand sequence
Negative control		GAAATGTACTGCGTGGAGA	TCTCCACGCAGTACATTTC
cTTN1	1	CCACCCAAAGAACCTCCAA	TTGGAGGTTCTTTGGGTGG
	2	CCCAAAGAACCTCCAAAGT	ACTTTGGAGGTTCTTTGGG
RBM20		CCCAGAGGGAGAGGGACAT	ATGTCCCTCTCCCTCTGGG
SRSF10		GTCCAAGAGGATTTGCTTA	TAAGCAAATCCTCTTGGAC

For all targeted RNAs we depict the exact RNA targeted sequence and the active antisense strand formed from the shRNA sequences.

Supplemental Table III. Antibodies

Gene	Supplier	Species	Dilution	Cat number
SRSF10 (FUSIP1)	Invitrogen	Rabbit	1:200 WB<br>5 µg RIP	PA5-62846
RBM20	Eurogentec custom Antibodies	Rabbit	1:100 ICC	Custom made
RBM20	Atlas antibodies	Rabbit	1:100 immunoFISH	HPA035806
cTnI	HyTest	Goat	1:400 ICC	4T21/2
Calnexin	Santa Cruz	Mouse	1:1500 WB	sc-23954
α-actinin	Epitomics	Rabbit	1:500 ICC	2310-1
DsRed	Santa Cruz	Mouse	1:250 ICC	Sc-390909
Flag Tag	Agilent	Mouse	5 µg RIP	200472
DAPI	Sigma		1:5000	D9542
FITC	Invitrogen	Rabbit	1:600 immunoFISH	A21253
Annexin V ATTO-488	Adipogen	E.coli	1:200 FACS	AG-40B-0005TD-T100
ECL HRP anti-mouse	GE healthcare	Sheep	1:5000 WB	NA9310V
ECL HRP anti-rabbit	GE healthcare	Sheep	1:5000 WB	NA9340
Alexa Fluor 647 anti-Rabbit	Invitrogen	Goat	1:250 ICC	A-21244
Alexa Fluor 647 Anti-Rabbit	Invitrogen	Donkey	1:250 ICC	A-31573
Alexa fluor 568 Anti-mouse	Invitrogen	Donkey	1:250 ICC	A10037
Alexa fluor 488 Anti-goat	Invitrogen	Donkey	1:250 ICC	A32814
Alexa fluor 488 Anti-rabbit	Invitrogen	Goat	1:250 ICC	A32731
Alexa fluor 568 Anti-mouse	Invitrogen	Goat	1:250 ICC	A-11031

This table contains all the details of the antibodies used in this study and for which experiments they are used (WB: Western Blot; RIP: RNA immunoprecipitation; ICC: immunocytochemistry; immunoFISH: immuno and fluorescent in situ hybridization; FACS: Fluorescence-Activited Cell Sorting).

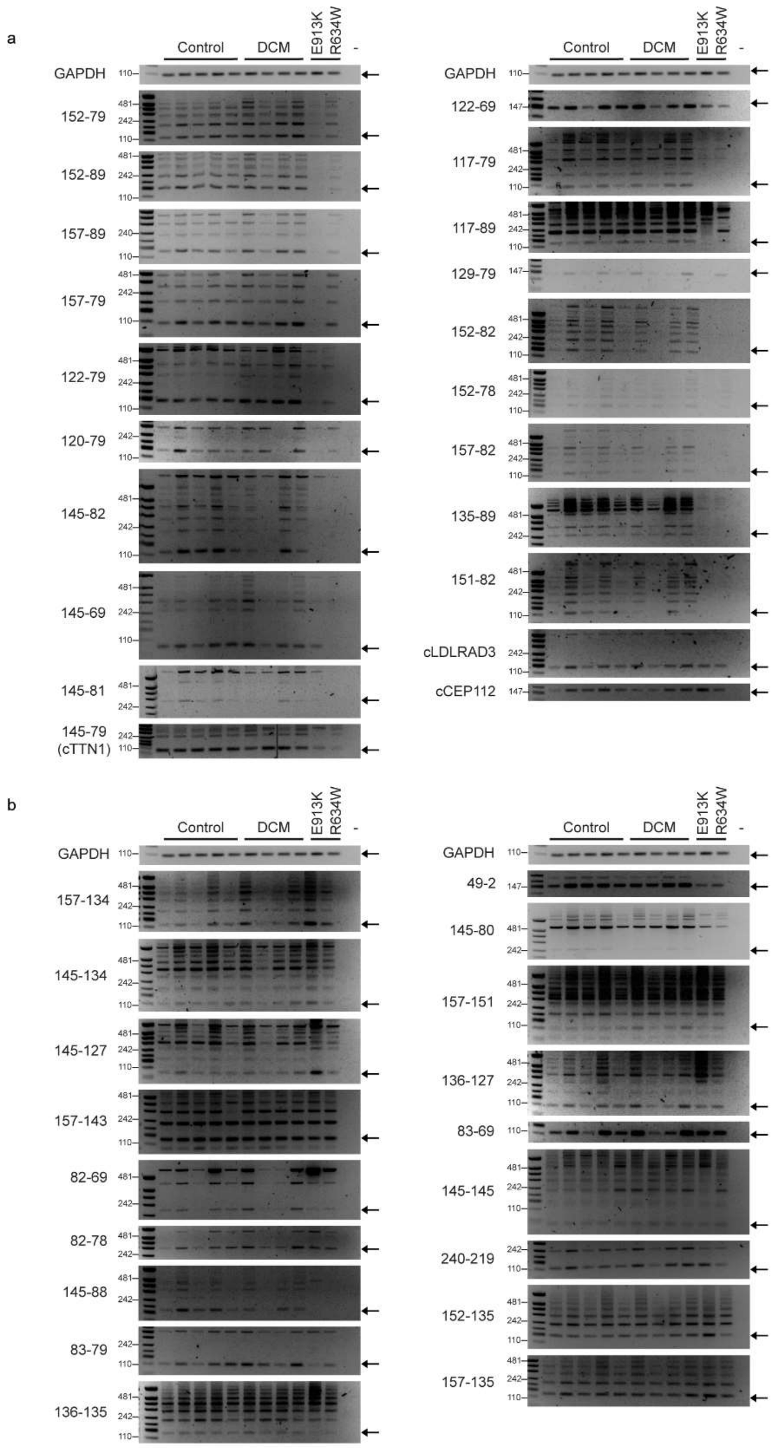

Supplemental Figure I. RBM20 dependent TTN-derived circRNAs contain the 8 nt AAAGAACC motif

RT-PCR of circRNAs containing the motif derived from *TTN*, *LDLRAD3* and *CEP11*2 (a) and circRNAs not containing the motif derived from *TTN* (b) in human healthy hearts, hearts from dilated cardiomyopathy (non-*RBM20* mutation carriers) and *RBM20* mutation carriers (E913K/R634W). Arrows indicated the sequence validated product of the detected backsplice junction.

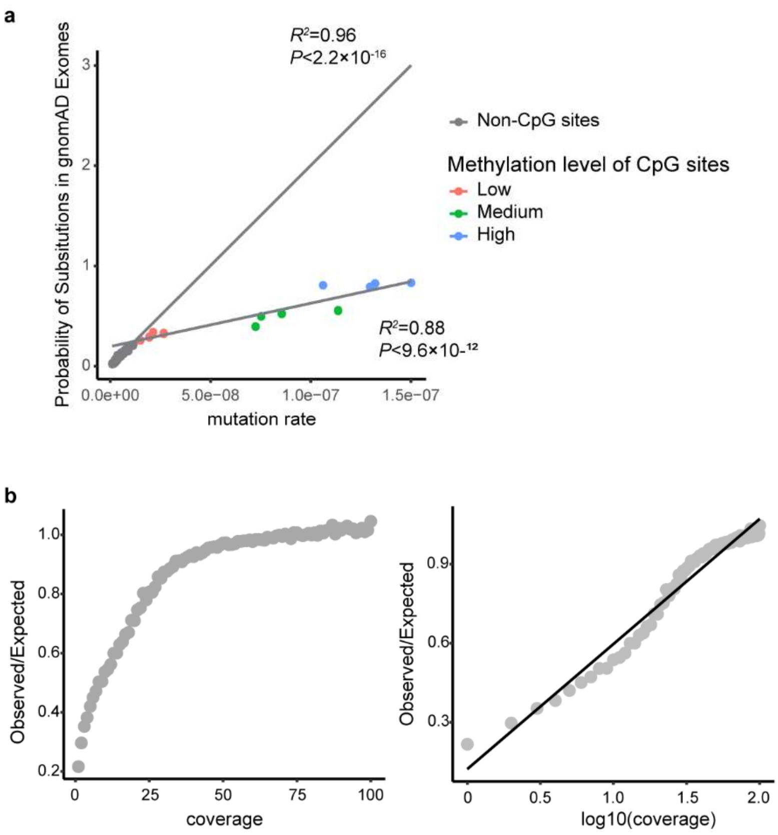

Supplemental Figure II. Calibration of the genetic constraint model

a) Two linear regression models (for CpG and non-CpG sites) were fitted to predict the proportions of neutral substitutions within the 125,478 exomes in gnomAD based on the baseline mutation rates. b) A linear model is fitted to predict the Observed/Expected ratios given a sequencing coverage after transforming it on a log10 scale. The predicted Observed/Expected ratios are used as correction factors to adjust the expected number of variants at low-coverage (median depth <40) sites.

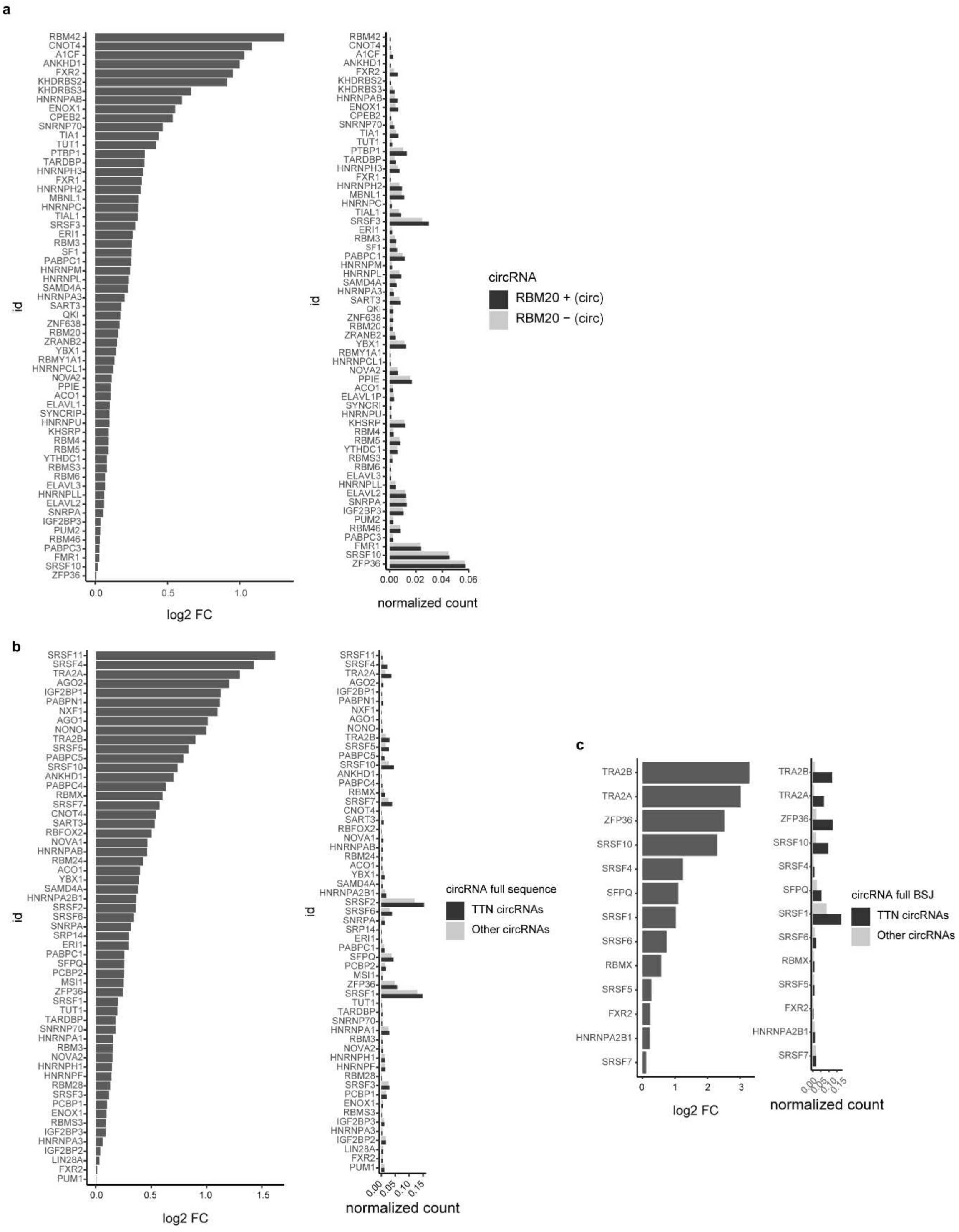

Supplemental Figure III. RNA binding protein analysis of circRNAs

Comparison of detected RNA binding protein motifs within the full circRNA sequence of 13 RBM20 dependent and 9 independent circRNAs (a), between the full circRNA sequence of 46 *TTN*-derived and 1412 non-*TTN* derived circRNAs (b), and between the backsplice junctions of 46 *TTN*-derived and 1412 non-*TTN* derived circRNAs (c).

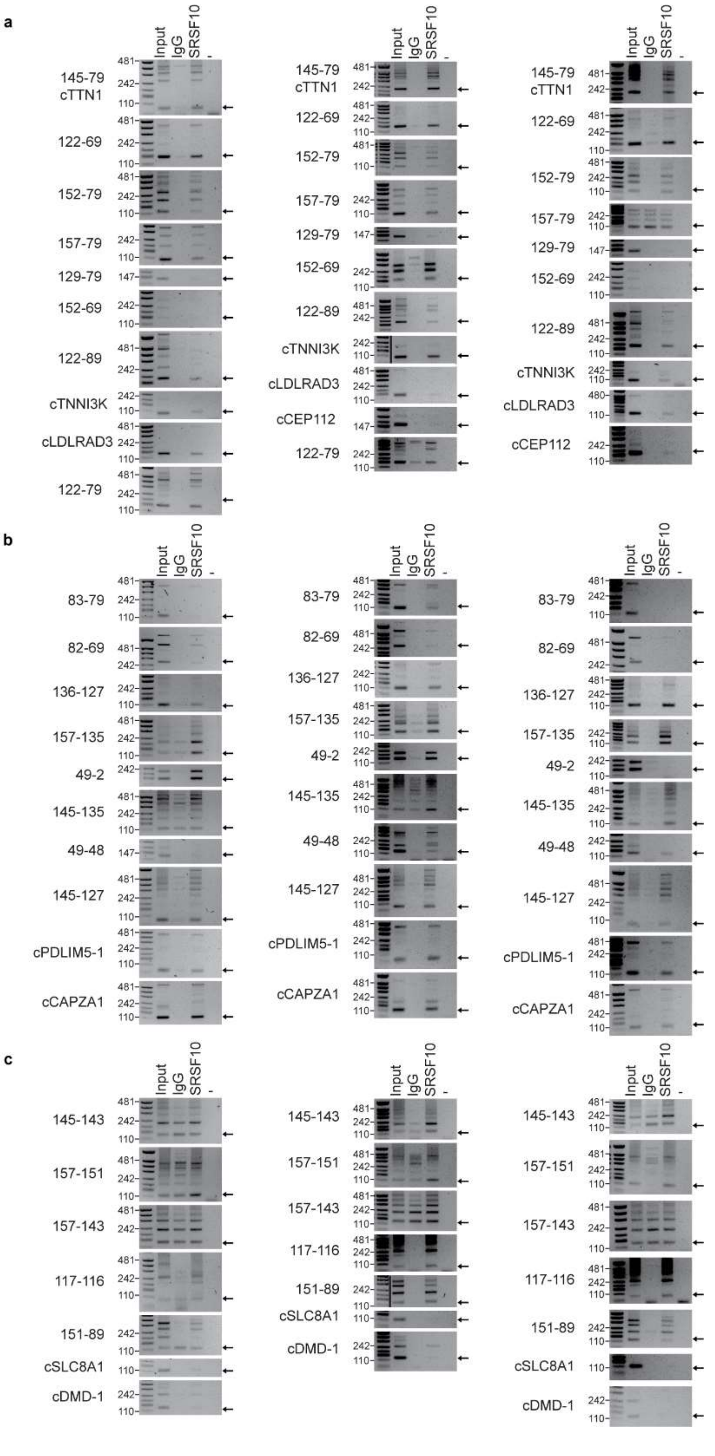

Supplemental Figure IV. Repeats of SRSF10 RNA immunoprecipitation experiments

RT-PCR based detection of circRNAs with the motif in their backsplice junction (a), full circRNA sequence (b) or without any motif (c) after immunoprecipitation based on endogenous SRSF10 in hiPSC-CM. The first column in all panels represents a direct repeat of figure 1f, and in the second and third column slight adaptations to the protocol were made, 1% BSA blocking of the beads and added UREA to the washings (1M for column 2 and 3M for column 3). cTTN1 was detected with 2 different sequence-verified primer-sets.

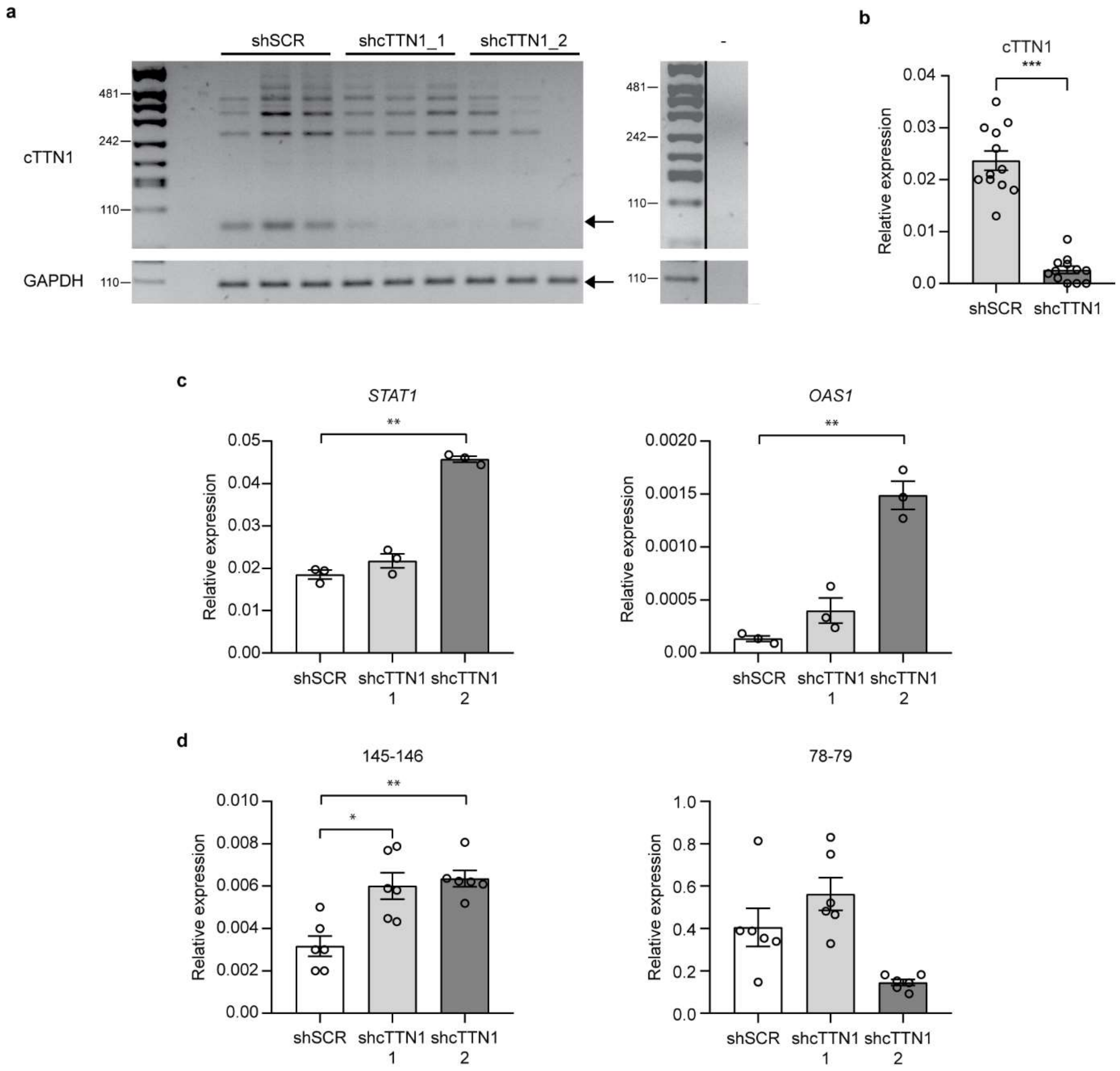

Supplemental Figure V. Validation of shRNAs targeting the cTTN1 backsplice junction

Knockdown of cTTN1 after shRNA transductions in hiPSC-CMs for 2 shRNAs detected by RT-PCR (a) or the eventual used shRNA1 by taqman based qRT-PCR (b, n=12, 4 differentiations). c) qRT-PCR for the interferon responsive genes *STAT1* and *OAS1* (n=3, 1 differentiation) and d) for the linear *TTN* transcript based on the linear junctions of the exons used for backsplicing to create cTTN1 (n=6, 2 differentiations). P-values *<0.05; **<0.025; ***<0.001; error bars SEM.

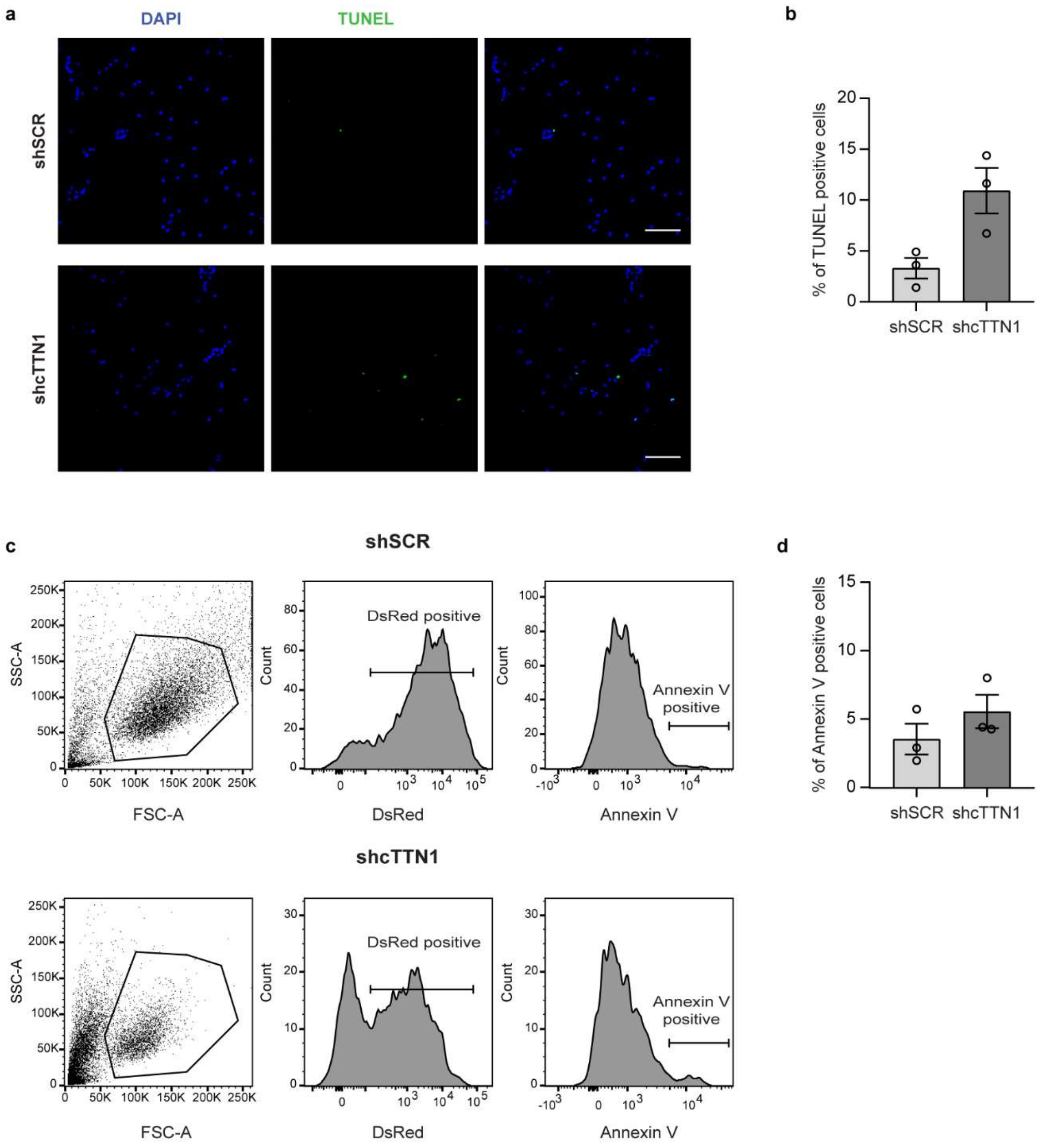

Supplemental Figure VI. Apoptosis after knockdown of cTTN1

Representative pictures (a) and quantification of percentage of TUNEL positive cells (b) after specific knockdown of cTTN1 by TUNEL staining (n=3 derived from 5-7 randomly selected areas per differentiation). c) Representative example of flow cytometry analysis showing the selected living cells, the DsRED positive cells and the Annexin V positive cells within this population. d) shows the apoptosis rate derived from the flow cytometry, which is calculated as the percentage of Annexin V positive cells within the DsRed positive cell population (n=3 differentiations. Error bars SEM.

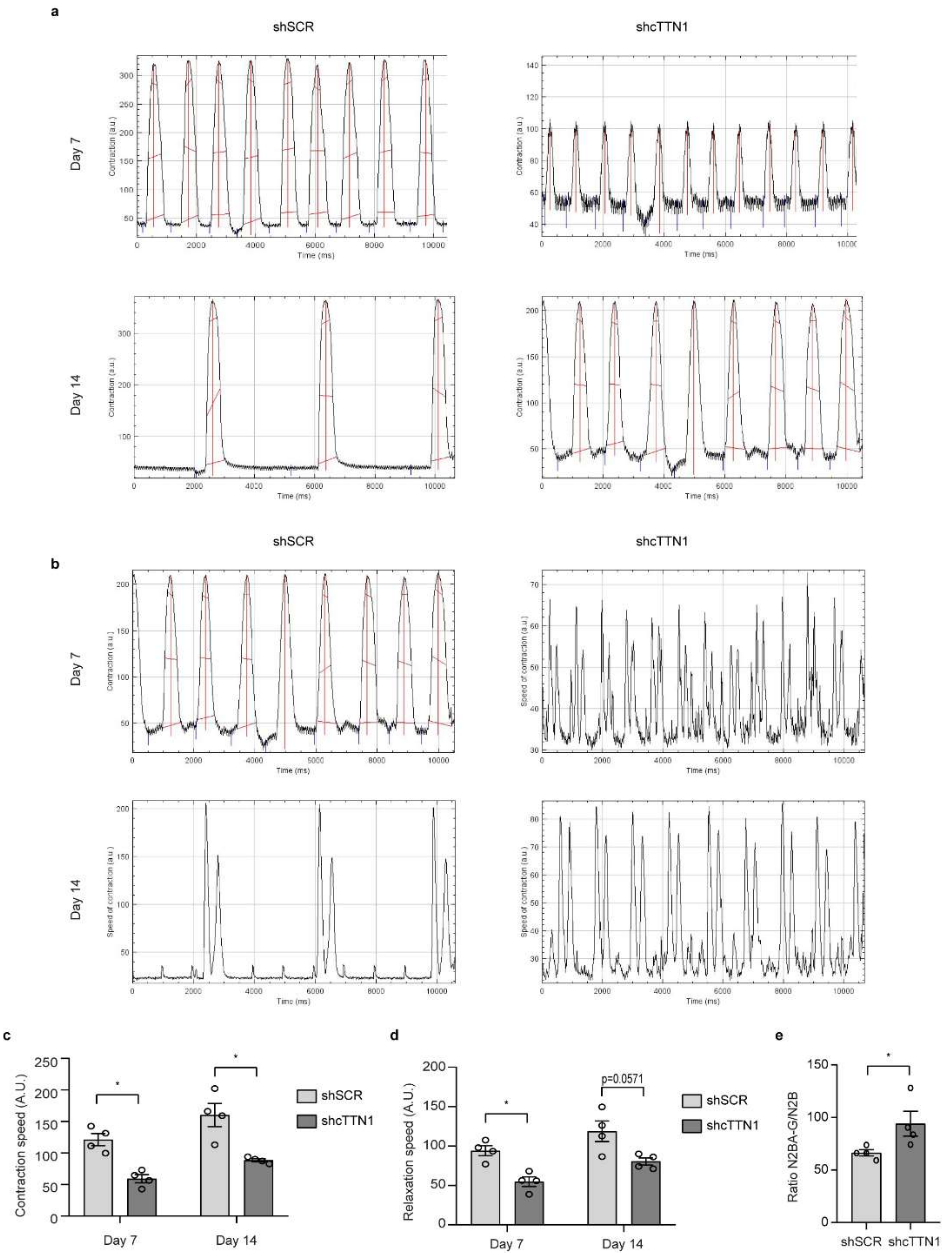

Supplemental Figure VII. Engineered heart tissue contraction after loss of cTTN1

Representative contraction profiles from the negative control (SCR) and cTTN1 knockdown EHT groups at 7 and 14 days after generation of the EHTs (a). Black line is contraction profile; vertical red line indicates where contractions was identified in the video; horizontal red line indicates percentage of transient calculated (10%, 50%, 90%). b) Representative contraction speed profile of negative control and cTTN1 knockdown EHTs at 7 and 14 days after generation of the EHTs. 1st peak corresponds to contraction, 2nd peak corresponds to relaxation. c) contraction and d) relaxation speed at 7 and 14 days after generation of EHTs. e) The ratio between *TTN* splice isoforms N2BA-G and N2B detected in the EHTs. N=4 from 3 differentiations; p-value *<0.05; error bars SEM.

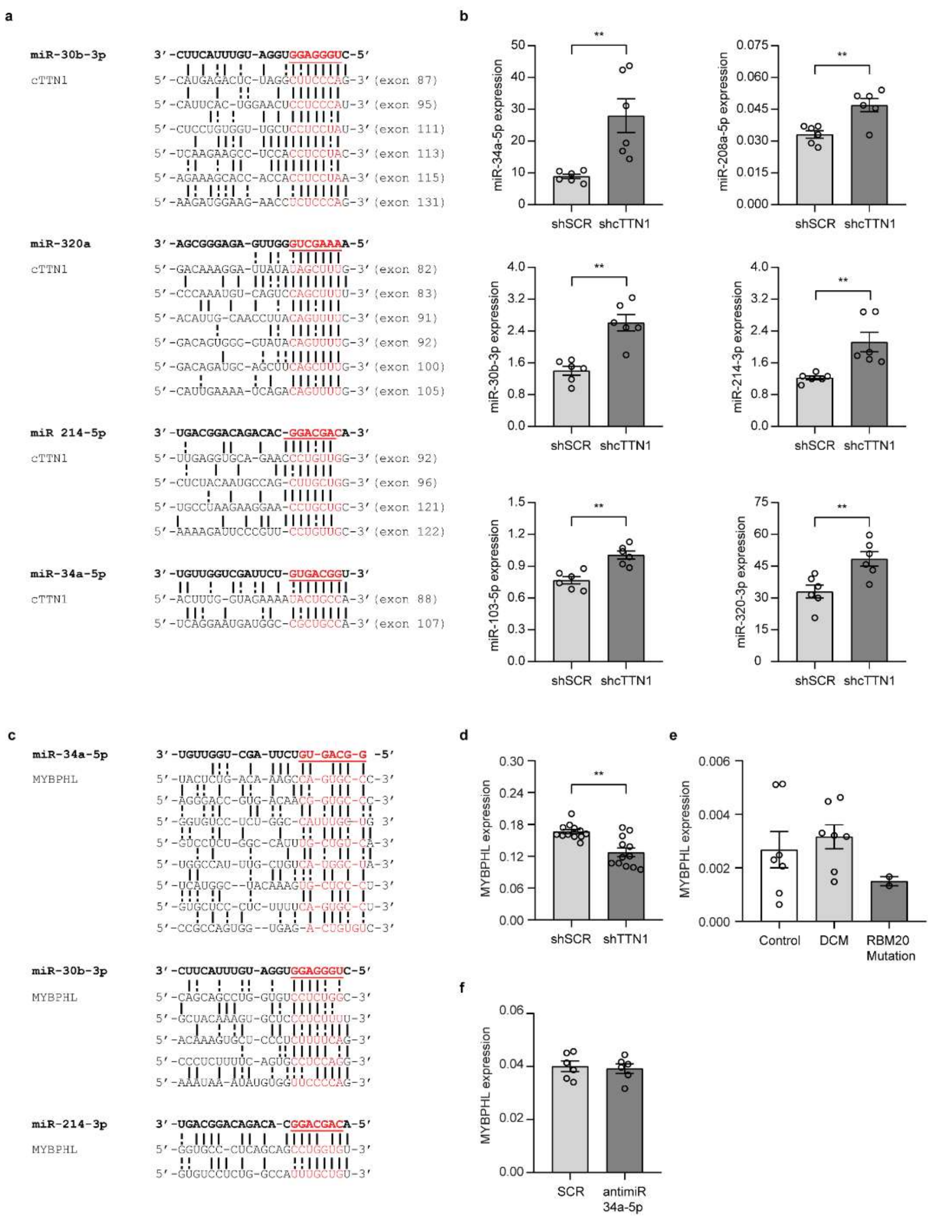

Supplemental figure VIII. cTTN1 sponges miRNAs to regulate MYPBHL expression

a) Predicted miRNA binding site reconstruction of miRNAs predicted to bind the cTTN1 sequence with highest stringency cutoffs. The seed region is depicted in red. b) qRT-PCR of miRNAs predicted to bind cTTN1 after loss of cTTN1 in hiPSC-CM (n=6;2 differentiations). c) Predicted miRNA binding sites reconstruction of miRNAs predicted to bind the 3´UTR of MYBPHL with all stringency cutoffs calculated in Supplemental Excel File VI. The seed region is depicted in red. d) qRT-PCR of *MYBPHL* after loss of cTTN1 in hiPSC-CMs (n=12;4 differentiations) and in (e) human hearts of controls (n=7), DCM patients (n=7) and DCM patients due to an *RBM20* mutation (n=2). f) qRT-PCR of *MYBPHL* after loss of miR-34 by antimiR transfection in hiPSC-CMs (n=6;2 differentiations). P-values **<0.025; error bars SEM.

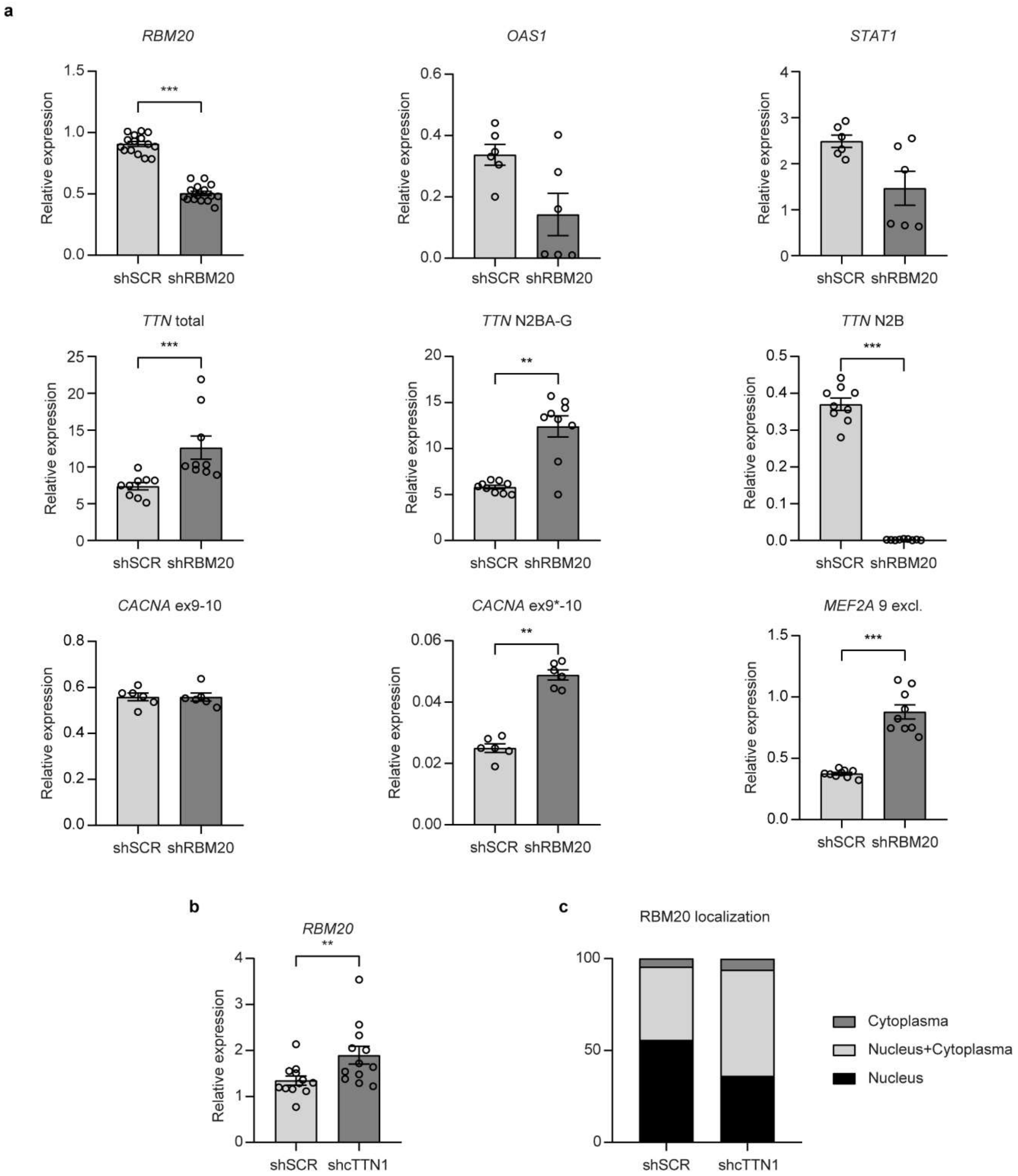

Supplemental Figure IX. Validation of shRNAs targeting RBM20

a) qRT-PCR after shRNA based inhibition of *RBM20* in hiPSC-CM for *RBM20* itself (n=15;5 differentiations), the interferon responsive genes *OAS1* and *STAT1* (n=6;2 differentiations), and splicing isoforms of *TTN* (n=9;3 differentiations), *CACNA1C* (n=6;2 differentiations) and *MEF2A* (n=9;3 differentiations). b) qRT-PCR of *RBM20* after loss of cTTN1 in hiPSC-CM (n=12;4 differentiations). P-values qRT-PCRs **<0.025; ***<0.001; error bars SEM c) Quantification of RBM20 localization after loss of cTTN1 based on the immunoFISH experiment, where a different antibody is used as in the immunocytochemistry (n=181 shSCR and 83 shcTTN1; 1 differentiation, p-value chi-square <0.001).

Supplemental Video legends

Supplemental Video I: Video of a contracting EHT after transduction with the SCR negative control shRNA

Supplemental Video II: Video of a contracting EHT after transduction with the shRNA directed against cTTN1

Supplemental Excel File legends

Supplemental Excel File I: RNA binding protein analysis of RBM20 dependent vs independent circRNAs within TTN

CircRNAprofiler output of the RNA binding protein analysis, containing the coordinates of the circRNAs analysed, the detected motifs in backsplice junction and full circRNA sequences and the comparison between RBM20 dependent and independent circRNAs for the SRSF10 binding motifs.

Supplemental Excel File II: RNA binding protein analysis of cTTN1, all highly expressed TTN circRNAs and linear TTN mRNA transcript

CircRNAprofiler output of the RNA binding protein analysis, containing the coordinates of the *TTN*-derived circRNAs analysed, the detected motifs in backsplice junctions and full circRNA sequences of these circRNAs, the detected motifs specifically in cTTN1 and the detected motifs in the linear mRNA transcript including all 363 *TTN* exons. This table also contains the comparison of SRSF10 binding motifs between *TTN* and non-*TTN*-derived circRNAs and between the *TTN*-derived circRNAs and the linear *TTN* mRNA transcript.

Supplemental Excel File III: CircAST output based on RNAseq of 3 RNAseR treated human myocardial samples

This table shows the included exons in circRNAs within the genomic region spanning exon 79 to 145, which comprises the cTTN1 region and gives an indication which exons could be included in cTTN1. Given are the exons detected in the 3 separate myocardial samples and the exons excluded from circRNAs in all 3 samples.

Supplemental Excel File IV: RNA sequencing analysis after cTTN1 inhibition

This table contains the output of the differential expression and exon usage analysis after shRNA-mediated knockdown of cTTN1 in hiPSC-CM. Furthermore, it contains the results of the pathway analysis based on the differential expressed genes or exons detected in the first analysis (cut-offs p_{adj} ≤0.05 and log2FC ≥1).

Supplemental Excel File V: Predicted miRNA binding sites within cTTN1

Results of miRNA binding site prediction by circRNAProfiler for all exons within the cTTN1 genomic region. Shown are the miRNAs included in the analysis after filtering on cardiac expression and the predicted binding sites for these miRNAs, where all the information is included to construct the actual binding sites. The different sheets include miRNA binding sites based on different stringencies: sheet 1 totalMatches=7, maxNonCanonicalMatches=0; sheet 2 totalMatches=7, maxNonCanonical Matches=1; sheet 3 totalMatches=6, maxNon CanonicalMatches=2.

Supplemental Excel File VI: Predicted miRNA binding sites within MYBPHL 3'UTR

Results of miRNA binding site prediction by circRNAProfiler for the 3'UTR of *MYBPHL*. Shown are the miRNAs included in the analysis after filtering on cardiac expression and the predicted binding sites for these miRNAs, where all the information is included to construct the actual binding sites. The different sheets include miRNA binding sites based on different stringencies: sheet 1 totalMatches=7, maxNonCanonicalMatches=0; sheet 2 totalMatches=7, maxNonCanonical Matches=1; sheet 3 totalMatches=6, maxNonCanonical Matches=2.
